# Chronic Post-Surgical Pain at 3 Months After Breast Cancer Surgery: Incidence, Predictors, and an Internally Validated Risk Model

**DOI:** 10.3390/cancers18152433

**Published:** 2026-07-29

**Authors:** Abdelilah Ghannam, Mohammed Anass Majbar, Youssef Motiaa, Halima Abahssain, Aiman El Fassi, Oussama Ssouni, Yassine El Bouazizi, Zakaria Houssain Belkhadir, Brahim Elahmadi, Amine Souadka

**Affiliations:** 1 Faculté de Médecine et de Pharmacie de Rabat, Université Mohammed V de Rabat, Impasse Souissi, Rabat 10100, Morocco; a.majbar@um5r.ac.ma (M.A.M.); o.ssouni@um5r.ac.ma (O.S.); z.belkhadir@um5r.ac.ma (Z.H.B.); brahimelahmadi@gmail.com (B.E.); a.souadka@um5r.ac.ma (A.S.); 2 Centre D’Etudes Doctorales Sciences de la Vie et de la Santé, Faculté de Médecine et de Pharmacie de Rabat, Université Mohammed V de Rabat, Rabat 10000, Morocco; elbouazizi.yassine@gmail.com; 3 Anesthesia & Intensive Care Department, National Institute of Oncology, CHU Ibn Sina, Rabat 10100, Morocco; ayman.elfassi00@gmail.com; 4 Surgical Oncology Department, National Institute of Oncology, CHU Ibn Sina, Rabat 10100, Morocco; 5 Equipe de Recherche en Oncologie Translationnelle (EROT), Faculté de Médecine et de Pharmacie de Rabat, Université Mohammed V de Rabat, Rabat 10100, Morocco; 6 Faculty of Medicine and Pharmacy of Tangier, Abdelmalek Essaadi University, Tangier 90000, Morocco; youssefmotiaa@gmail.com; 7 Oncology and Medical Specialties Department, Valenciennes General Hospital, 59300 Valenciennes, France; abahssainhalima@gmail.com

**Keywords:** breast cancer surgery, chronic post-surgical pain, pain catastrophising, regional anaesthesia, prediction model, mediation analysis, ketoprofen, MENA region

## Abstract

**Background/Objectives**: Chronic post-surgical pain (CPSP) affects 25–60% of women after breast cancer surgery. Prospective data from the Middle East and North Africa (MENA) region remain scarce. This study estimated 3-month CPSP incidence in a Moroccan cohort, developed two internally validated prediction models, and quantified the mediated effects of regional anaesthesia and ketamine through prevention of severe acute postoperative pain. **Methods**: Prospective cohort of 862 women undergoing curative breast cancer surgery (Institut National d’Oncologie, Rabat; NCT04676438). CPSP was defined as a visual analogue scale (VAS) ≥ 3/10 AND Brief Pain Inventory (BPI) interference ≥ 3/10 at three months. Two logistic regression models were developed (Model A: 7 preoperative variables; Model B: 11 perioperative variables) and internally validated by 1000-bootstrap optimism correction. Counterfactual mediation analysis quantified effects mediated through severe acute pain. **Results**: CPSP incidence was 39.1% (95% confidence interval (CI) 35.9–42.4%), with 22.6% neuropathic-predominant. Model A showed limited discrimination (area under the curve (AUC) 0.58), with pain catastrophising as the dominant predictor. Model B demonstrated superior performance (AUC 0.694; Brier score 0.209) with net clinical benefit across the 15–55% range of clinically plausible decision thresholds. In counterfactual mediation analysis, an estimated 58.2% and 53.7% of the RA and ketamine effects, respectively, were mediated through severe acute pain, under the framework’s no-unmeasured-confounding assumption. Perioperative ketoprofen independently reduced chronification risk (adjusted odds ratio (aOR) 0.66). **Conclusions**: In this single-centre Moroccan cohort, 39% of women developed CPSP at three months. Two validated models enable risk stratification at distinct clinical decision points. Regional anaesthesia and ketamine were associated with lower CPSP risk, in a pattern consistent with mediation through prevention of severe acute pain under the model’s assumptions; ketoprofen was independently associated with lower chronification risk. These observational findings are hypothesis-generating and require confirmation before informing changes to analgesic protocols.

## Simple Summary

Chronic post-surgical pain (CPSP) affects up to 60% of women after breast cancer surgery but remains poorly studied in the Middle East and North Africa (MENA) region. In this prospective study of 862 patients at the Institut National d’Oncologie in Rabat, Morocco, 39% developed CPSP at three months. We developed and validated two prediction models usable at different time points: one preoperative (based on psychological factors), and one at 48 h post-surgery (adding pain intensity, neuropathic signs, and analgesic profile). We found, to our knowledge, for the first time in this population, that regional anaesthesia and intravenous ketamine were associated with lower chronic pain risk, in a pattern consistent with mediation through prevention of severe acute postoperative pain. Protocol-recommended ketoprofen administration from surgical onset was independently associated with less pain chronification beyond its effect on acute pain, a pattern that may reflect a direct anti-sensitization effect but requires confirmation in randomised studies. These findings offer practical tools for risk stratification and targeted CPSP prevention in breast cancer patients and provide rare prospective data from Morocco for the under-represented MENA region.

## 1. Introduction

Breast cancer is the most common malignancy in women worldwide [1], with an incidence of 49.2 new cases per 100,000 woman-years in the Moroccan Rabat registry [2]. Advances in treatment, with 5-year survival exceeding 80% in high-income countries, have shifted the clinical focus towards post-therapeutic quality of life [3], in which chronic post-surgical pain (CPSP) plays a central role.

CPSP is defined by the International Association for the Study of Pain (IASP) classification, incorporated into the International Classification of Diseases, 11th Revision (ICD-11; code MG30.21), as pain persisting beyond three months after a surgical procedure, localised in the operative field or its innervation territory, and not attributable to another cause [4]. It affects 25% to 60% of patients after breast surgery [5,6], with well-documented functional, social, and psychological consequences [7,8], and a predominant neuropathic component.

Published risk factors are heterogeneous, and predictive models remain rare, with limited internal validation, largely derived from North American or European cohorts [9,10,11,12]. Prospective data from the Middle East and North Africa (MENA) region are scarce [13,14], despite differences in oncological management, analgesic resources, and catastrophising prevalence. Mechanistically, the protective effects of regional anaesthesia (RA) and intravenous ketamine, well documented in the acute phase [15,16], remain debated for pain chronification, and their effects, potentially mediated through prevention of severe acute pain, have, to our knowledge, never been formally quantified in this population.

This study pursues three objectives: (i) to estimate CPSP incidence at three months after curative breast cancer surgery in a Moroccan cohort, using a strict composite definition in accordance with IASP and Initiative on Methods, Measurement, and Pain Assessment In Clinical Trials (IMMPACT) recommendations; (ii) to identify independent predictors and develop two complementary, internally validated clinical risk models following Transparent Reporting Of A Multivariable Prediction Model For Individual Prognosis Or Diagnosis (TRIPOD) recommendations, one for use at the preanaesthetic consultation (Model A) and one at postoperative day 2 (Model B); and (iii) to quantify, via counterfactual mediation analysis, the proportion of the RA and intraoperative ketamine effects mediated through severe acute postoperative pain.

## 2. Materials and Methods

### 2.1. Study Design and Ethics

Prospective, single-centre, observational cohort study conducted at the Institut National d’Oncologie of Rabat (INO), Ibn Sina University Hospital, between December 2020 and March 2023. The protocol was approved by the Research Ethics Committee for Biomedical Research of the Faculty of Medicine and Pharmacy of Rabat (ref. C52-20, 8 December 2020) and prospectively registered on ClinicalTrials.gov (NCT04676438). This report follows the Strengthening the Reporting of Observational Studies in Epidemiology (STROBE) and TRIPOD recommendations; checklists are provided as Supplementary Materials S2 and S3. This was an observational cohort study: all anaesthetic and analgesic decisions were taken by the clinical team as part of routine perioperative care, and no treatment was allocated to patients for research purposes. The Ethics Committee approved the consecutive enrolment of all eligible patients over the predefined recruitment period (December 2020–March 2023) rather than a fixed numerical target. Study-specific procedures were limited to validated questionnaires and a single non-invasive bedside sensory (pinprick) assessment, entailing minimal incremental risk beyond standard care.

### 2.2. Study Population

Eligible patients were women aged ≥ 18 years scheduled for curative-intent breast surgery for histologically confirmed cancer, who provided written informed consent. Stage IV patients were eligible when surgery was part of a local curative strategy following effective systemic cytoreduction (documented partial or complete response); although this is non-standard in CPSP studies, it reflects the real-world practice at the Institut National d’Oncologie, where a proportion of stage IV patients undergo curative-intent local surgery after systemic response. To assess the impact of this inclusion, a pre-specified exclusion sensitivity analysis (analysis #4, Table S3) was performed; results were consistent with the primary analysis (severe acute pain aOR 3.48, ketoprofen aOR 0.68), supporting the robustness of findings in the standard stage I–III population. Exclusion criteria were non-elective or non-oncological surgery and any pre-existing extra-mammary chronic pain not formally documented.

### 2.3. Anaesthetic and Analgesic Protocol

General anaesthesia was conducted according to standardised institutional protocols, unchanged throughout the inclusion period, by a single anaesthetic team.

Protocol-recommended baseline analgesia. Paracetamol (1 g IV) was administered to all patients before anaesthetic emergence, then continued at 1 g every 8 h from H4 onwards. Ketoprofen (100 mg IV) was recommended at the time of surgical incision, when available and in the absence of contraindications (renal impairment, peptic ulcer, and thrombocytopenia < 100 × 10^9^/L, non-steroidal anti-inflammatory drug (NSAID) allergy), then every 12 h over H0–H48.

Team-discretion analgesia. The remaining peri- and postoperative analgesic protocol was determined by the anaesthetic team based on clinical context: (a) RA via ultrasound-guided thoracic fascial-plane block; (b) intraoperative IV ketamine (bolus 0.15–0.5 mg·kg^−1^ then infusion 0.1–0.2 mg·kg^−1^·h^−1^ until 30 min before end); (c) IV lidocaine (bolus 1.5 mg·kg^−1^ then infusion 1.5 mg·kg^−1^·h^−1^); (d) opioid-free anaesthesia (OFA) at the anaesthetist’s discretion. Nefopam 20 mg IV every 6 h as first-line rescue (visual analogue scale (VAS) > 4); subcutaneous morphine 0.1 mg·kg^−1^ as second-line rescue (VAS > 6 or nefopam failure). Full protocol details, including the institutional regional anaesthesia allocation algorithm (Table S5), are provided in Supplementary S13.

### 2.4. Data Collection and Candidate Predictors

Preoperative variables. Demographics (age, body mass index (BMI), menopausal status), comorbidities (diabetes, hypertension, and American Society of Anesthesiologists (ASA) score), surgical and pain history, oncological data (tumour–node–metastasis (TNM) stage, molecular subtype, neoadjuvant chemotherapy). Pain catastrophising was assessed using the Pain Catastrophising Scale (PCS, validated Arabic version [17]), threshold ≥ 30, and as a continuous variable (per +1 Standard Deviation (SD)). Anxiety and depression were measured using the Hospital Anxiety and Depression Scale (HADS) anxiety and depression subscales (HADS-A and HADS-D; validated Arabic version [18]), threshold ≥ 8.

Intraoperative variables. Surgery type (mastectomy vs. breast-conserving surgery (BCS)), axillary lymph node dissection (ALND) vs. sentinel lymph node biopsy (SLNB), documented preservation of the intercostobrachial nerve (ICBN), anaesthetic technique, RA (presence/absence and block type), IV ketamine, IV lidocaine, and opioid-free anaesthesia (OFA).

Early postoperative variables. VAS at rest and on movement at six time points (Post-anaesthesia care unit (PACU), H3, H6, H12, H24, H48). Severe acute postoperative pain was defined as VAS ≥ 5/10 at two or more consecutive time points within the H0–H24 window, preserving the temporal causal order required for mediation analysis (mediator precedes Day-90 outcome; avoids overlap with Douleur Neuropathique en 4 Questions (DN4) score measured at H48). Ketoprofen administration was recorded as a binary variable. Nefopam and morphine rescue use were documented separately. DN4 at H48 (validated Arabic version [19], positivity threshold ≥ 4/10) was administered as an early neuropathic marker. Peri-incisional hyperalgesia was assessed using a standardised pinprick test (single-use Neurotip^®^, Owen Mumford Ltd., Oxford, UK) applied to the peri-incisional area before ward discharge (H48).

### 2.5. Primary Outcome

CPSP at 3 months was defined using a strict composite criterion: (i) pain localised in the operative field or intercostobrachial territory, persisting ≥ 3 months, unexplained by tumour recurrence or documented infection; (ii) mean VAS ≥ 3/10 over the previous 7 days [20]; and (iii) at least one Brief Pain Inventory (BPI) Interference item ≥ 3/10 [21]. Assessment at day 90 (±14 days) was conducted by a trained physician, independent of perioperative care and blinded to exposure groups, via in-person algology consultation (*n* = 782; 90.7%) or structured telephone interview (*n* = 80; 9.3%). Deaths and recurrences documented before day 90 (*n* = 7) were classified as non-evaluable and excluded from the primary analysis.

### 2.6. Sample Size

Calculated using the Riley et al. method [22] for clinical prediction model development (R package pmsampsize): expected prevalence 35%, target shrinkage ≥ 0.9, anticipated Cox-Snell R^2^ 0.15, intercept error margin ≤ 0.05. For Model A (7 preoperative candidate predictors), the minimum required sample size was 245 patients (event per variable (EPV) target ≥ 20); for Model B (11 pre-specified candidate predictors), the minimum was 385 patients. The analysed cohort of 862 patients (337 events, CPSP incidence 39.1%) yields an EPV of 48.1 for Model A and 22.1 for Model B, both substantially exceeding the recommended thresholds [22] and ensuring a shrinkage factor ≥ 0.93. These values represent the minimum sample size required for reliable model development and internal validation, not a recruitment ceiling. In accordance with the approved protocol, all consecutive eligible patients during the predefined study period were enrolled; the resulting cohort (*N* = 862) therefore exceeds the calculated minima. This larger sample increases the number of events per variable, reduces optimism and overfitting, and enables the eight pre-specified sensitivity analyses and the 1000-iteration bootstrap internal validation reported below—an approach consistent with TRIPOD recommendations and with the observational nature of the study.

### 2.7. Statistical Analysis

Descriptive statistics. Continuous variables: median [Q1; Q3]; categorical variables: n (%). Comparisons: Mann–Whitney and χ^2^/Fisher tests; two-sided α = 0.05.

Missing data. Multiple imputation by chained equations (MICE, m = 20, R package mice v3.16, predictive mean matching for continuous variables and logistic regression for binary variables, seed 12345); estimates combined using Rubin’s rules [23,24]. The value of m = 20 was chosen to exceed the percentage of incomplete cases (48.6%), ensuring that sampling variability from the imputation step contributes less than 5% to total variance [24]. Complete-case analysis reported as sensitivity analysis #3.

Modelling strategy. Two complementary models. In accordance with TRIPOD recommendations and the pre-specified Statistical Analysis Plan (SAP v1.0, December 2020), two multivariable logistic regression models with clinically pre-specified predictors were developed without post hoc variable selection (Table S1): Model A (7 preoperative variables, for use at the preanaesthetic consultation) and Model B (11 pre-specified variables including perioperative and early postoperative markers, for use at postoperative day 2). The Variance Inflation Factor (VIF) quantifies multicollinearity among predictors: VIF = 1 indicates no collinearity, values 1–2 are acceptable, and VIF > 4 is a threshold requiring attention. All VIF values remained < 2 in both models. Linearity of continuous variables assessed by fractional polynomials.

Performance and internal validation. Discrimination: Area under the curve (AUC) (DeLong 95% confidence interval (CI)). Calibration: calibration intercept and slope, Brier score contextualised against the null-model Brier (*p* × (1 − *p*) = 0.238 for incidence 39.1%), and Locally Estimated Scatterplot Smoothing (LOESS) calibration plot. Internal validation: 1000 bootstrap iterations with optimism correction [25]. Clinical utility: Decision curve analysis (DCA) (R package rmda) [26].

Counterfactual mediation analysis. VanderWeele framework [27], R package mediation v4.5, 1000 bootstrap iterations. Mediator: severe acute pain (H0–H24). Natural direct effect (NDE), natural indirect effect (NIE), and proportion mediated are reported on both the odds ratio and risk-difference scales. E-values (R package EValue; [28]) reported for NDE robustness to unmeasured confounding. Adjusted confounders: age, BMI, TNM stage, surgery type, catastrophising, HADS-D, and ICBN preservation. Exposure × mediator interaction was tested and was non-significant (*p* > 0.30 for both exposures).

Pre-specified sensitivity analyses (8). (1) CPSP threshold VAS ≥ 4; (2) VAS ≥ 7; (3) complete cases only; (4) exclusion of stage IV; (5) tipping-point analysis [29]); (6) Least Absolute Shrinkage And Selection Operator (LASSO) alternative model; (7) mediator defined over H0–H48; (8) proportion mediated on the risk-difference scale. All analyses: R 4.3 (R Core Team, Vienna, Austria).

## 3. Results

### 3.1. Patient Flow and Analysed Population

**Figure 1:**
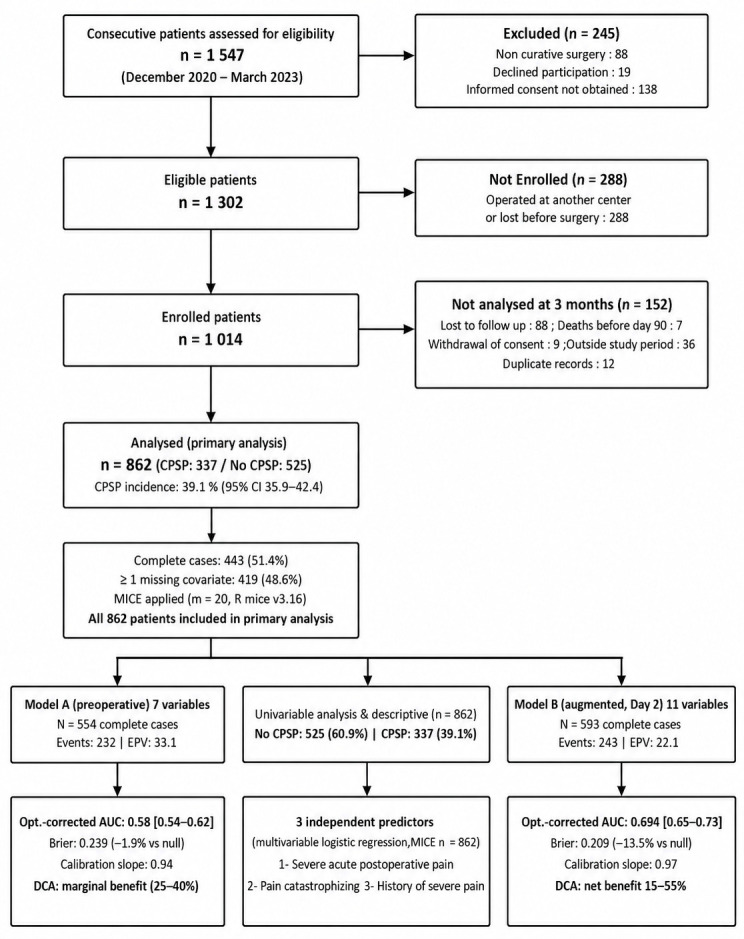
Strobe flowchart.

Of 1547 consecutive patients assessed for eligibility between December 2020 and March 2023, 245 were excluded at the eligibility stage (non-curative surgery *n* = 88; declined to participate *n* = 19; written informed consent not obtained *n* = 138), leaving 1302 eligible patients. Of these, 288 were not enrolled (operated at another centre or lost before surgery). Of the 1014 enrolled patients, 152 could not be analysed at 3 months (lost to follow-up *n* = 88; death before day 90 *n* = 7; withdrawal of consent *n* = 9; evaluated outside the study period *n* = 36; duplicate records identified *n* = 12), yielding 862 analysed patients (Figure 1). Day-90 follow-up was achieved by in-person consultation for 782 patients (90.7%) and by structured telephone interview for 80 (9.3%). The seven deaths documented before day 90 were classified as non-evaluable and excluded from the primary analysis; none were attributed to the study intervention.

### 3.2. Baseline Characteristics

Median age was 48 years [Q1: 40; Q3: 57] and median BMI 30.0 kg·m^−2^ [24.9; 35.8]. Mastectomy was performed in 594 patients (68.9%) and ALND in 576 (66.8%). Stage distribution: 0–I 42.3%, II 23.3%, III 11.7%, IV (with local curative surgery) 12.9%. RA was performed in 130 patients (15.1%), and intraoperative ketamine was administered in 171 (19.8%); ketoprofen was administered in 435 patients (50.5%). Demographic, psychological, and oncological characteristics are detailed in Table 1; surgical, anaesthetic, and pain data in Table 2.

None of the analysed patients had received adjuvant radiotherapy before the 3-month CPSP assessment.

**Table 1:** Demographic, psychological, and oncological characteristics according to CPSP status at 3 months.

Variable	Total (*N* = 862)	No CPSP (*n* = 525)	CPSP (*n* = 337)	*p*
**Demographic data**				
Age (years), median [Q1; Q3]	48 [40; 57]	48 [41; 57]	47 [40; 57]	0.452
BMI (kg·m^−2^), median [Q1; Q3]	30.0 [24.9; 35.8]	29.8 [25.0; 35.5]	30.1 [24.9; 36.2]	0.493
Menopausal (peri/post vs. pre), n (%)	436 (50.6%)	273 (52.0%)	163 (48.4%)	0.298
Diabetes, n (%)	109 (12.7%)	58 (11.1%)	39 (11.6%)	0.898
Hypertension, n (%)	124 (14.5%)	66 (12.7%)	43 (12.9%)	1
ASA score ≥ 3, n (%)	108 (12.5%)	64 (12.2%)	44 (13.1%)	0.788
**Psychological assessment**				
Pain catastrophising (PCS ≥ 30), n (%)	123 (15.0%)	61 (12.6%)	62 (18.4%)	0.029 *
Anxiety (HADS-A ≥ 8), n (%)	304 (37.6%)	186 (37.7%)	118 (37.3%)	0.971
History of severe pain, n (%)	96 (12.7%)	50 (10.8%)	46 (15.6%)	0.072
Prior breast surgery, n (%)	41 (4.9%)	21 (4.1%)	20 (6.2%)	0.239
**Oncological data**				
Stage 0–I, n (%)	365 (42.3%)	221 (42.1%)	144 (42.7%)	0.217
Stage II, n (%)	201 (23.3%)	127 (24.2%)	74 (22.0%)	—
Stage III, n (%)	101 (11.7%)	55 (10.5%)	46 (13.6%)	—
Stage IV (local curative surgery), n (%)	111 (12.9%)	66 (12.6%)	45 (13.4%)	—
Triple-negative, n (%)	171 (21.6%)	100 (20.8%)	71 (22.7%)	0.595
Neoadjuvant chemotherapy, n (%)	108 (12.5%)	63 (12.0%)	45 (13.4%)	0.631

Median [Q1; Q3] or n (%). * *p* < 0.05 (Mann–Whitney or χ^2^/Fisher). PCS: Pain Catastrophising Scale; HADS-A: Hospital Anxiety and Depression Scale—anxiety subscale; BMI: body mass index; ASA: American Society of Anesthesiologists.

**Table 2:** Surgical, anaesthetic, and pain data according to CPSP status at 3 months.

Variable	Total (*N* = 862)	No CPSP (*n* = 525)	CPSP (*n* = 337)	*p*
**Surgery**				
Mastectomy (vs. BCS), n (%)	594 (68.9%)	353 (67.2%)	241 (71.5%)	0.212
ALND (vs. SLNB), n (%)	576 (66.8%)	343 (65.3%)	233 (69.1%)	0.278
Documented ICBN preservation, n (%)	489 (56.7%)	318 (60.6%)	171 (50.7%)	0.005 **
Surgical duration (min), median [Q1; Q3]	95 [70; 130]	92 [70; 125]	100 [75; 140]	0.06
**Intraoperative anaesthesia**				
RA (ultrasound-guided thoracic fascial-plane block), n (%)	130 (15.1%)	90 (17.1%)	40 (11.9%)	0.044 *
Intraoperative IV ketamine, n (%)	171 (19.8%)	120 (22.9%)	51 (15.1%)	0.007 **
Intraoperative IV lidocaine, n (%)	98 (11.4%)	62 (11.8%)	36 (10.7%)	0.69
OFA (opioid-free anaesthesia), n (%)	175 (20.3%)	101 (19.2%)	74 (22.0%)	0.378
**Postoperative analgesia (H0–H48)**				
Ketoprofen administered (H0–H48), n (%)	435 (50.5%)	287 (54.7%)	148 (43.9%)	0.003 **
Nefopam rescue, n (%)	212 (24.6%)	121 (23.0%)	91 (27.0%)	0.217
Morphine rescue, n (%)	99 (11.5%)	57 (10.9%)	42 (12.5%)	0.54
**Acute pain and early neuropathic markers**				
VAS at PACU, median [Q1; Q3]	3 [2; 4]	3 [2; 4]	4 [2; 5]	<0.001 ***
VAS on movement at H3, median [Q1; Q3]	4 [3; 5]	4 [3; 5]	5 [3; 6]	<0.001 ***
Severe acute pain (VAS ≥ 5, ≥ 2 time points H0–H24), n (%)	360 (41.8%)	158 (30.1%)	202 (59.9%)	<0.001 ***
DN4 positive at H48 (score ≥ 4/10), n (%)	81 (10.4%)	36 (7.7%)	45 (14.5%)	0.003 **
Peri-incisional hyperalgesia (pinprick, Neurotip^®^), n (%)	137 (17.1%)	73 (15.0%)	64 (20.6%)	0.05

* *p* < 0.05; ** *p* < 0.01; *** *p* < 0.001. BCS: breast-conserving surgery; ALND: axillary lymph node dissection; SLNB: sentinel lymph node biopsy; ICBN: intercostobrachial nerve; RA: regional anaesthesia; OFA: opioid-free anaesthesia; PACU: post-anaesthesia care unit; VAS: visual analogue scale; DN4: Douleur Neuropathique 4 Questions.

### 3.3. Missing Data

Little’s Missing Completely At Random (MCAR) test did not reject the null hypothesis of absence of randomness (*p* = 0.22); a Missing At Random (MAR) mechanism was retained. Among the 30 baseline variables, 29 showed a standardised difference |d| < 0.10 between complete and incomplete observations; ICBN preservation alone reached |d| = 0.10 (Table S2). Imputation convergence was verified graphically (trace plots, 20 iterations). The highest individual missing-data rate was 12.3% for ‘history of severe pain’; all other variables were missing in < 5% of records. A detailed missing-data heatmap is provided in Figure S1.

### 3.4. CPSP Incidence at 3 Months

CPSP incidence at 3 months was 39.1% (337/862; 95% CI 35.9–42.4%). Mechanistic decomposition identified 142 patients (16.5%) with predominantly nociceptive pain (CPSP + DN4 < 4 at 3 months) and 195 (22.6%) with predominantly neuropathic pain (CPSP + DN4 ≥ 4 at 3 months). Among CPSP-positive patients, the most frequent pain sites were the scar region (74.2%), residual breast or mammary area (58.2%), axillary region (41.2%), and arm (32.3%). Restricting the analysis to the standard stage I–III population (*n* = 751) yielded a closely similar incidence of 38.9% (292/751; 95% CI 35.4–42.4%), indicating that inclusion of stage IV patients did not materially affect the headline estimate (consistent with pre-specified sensitivity analysis #4, Table S3).

### 3.5. Univariable Analyses

In univariable analysis, eight variables were significantly associated with CPSP (Table 3): severe acute pain (odds ratio (OR) 3.48 [2.61–4.63]; *p* < 0.001), DN4-positive at H48 (OR 2.04 [1.28–3.24]; *p* = 0.003), preoperative pain catastrophising (OR 1.56 [1.06–2.30]; *p* = 0.023), history of severe pain (OR 1.52 [0.99–2.33]; *p* = 0.057, trend), peri-incisional hyperalgesia (OR 1.47 [1.02–2.13]; *p* = 0.041), postoperative ketoprofen (OR 0.65 [0.49–0.86]; *p* = 0.002, protective), IV ketamine (OR 0.60 [0.42–0.86]; *p* = 0.006, protective), and RA (OR 0.65 [0.44–0.97]; *p* = 0.036, protective). Postoperative VAS pain trajectories stratified by CPSP status are illustrated in Figure S5. The unadjusted CPSP incidence by key predictor strata is shown in Figure S6; notably, the protective effect of ketoprofen is visible in the univariable analysis (32.8% vs. 44.9%; *p* = 0.003) and persists after full adjustment in Model B.

### 3.6. Model A—Preoperative Predictors

Model A (7 preoperative variables; *N* = 554 complete cases, 232 events, EPV = 33.1) demonstrated limited discrimination (optimism-corrected AUC 0.58; 95% CI 0.54–0.62; Brier score 0.239, and −1.9% improvement vs. null). The sole statistically significant independent predictor was preoperative pain catastrophising (adjusted odds ratio (aOR) 1.78 [1.13–2.81]; *p* = 0.013). History of severe pain (aOR 1.47 [0.89–2.44]; *p* = 0.134) and prior breast surgery (aOR 1.67 [0.78–3.60]; *p* = 0.189) showed trends. Oncological variables and anxiety did not contribute to discrimination. DCA showed marginal but positive net benefit over the 25–40% threshold range, supporting selective utility for targeting psychologically at-risk patients at the preanaesthetic consultation.

### 3.7. Model B—Augmented Early Postoperative Model

**Figure 2:**
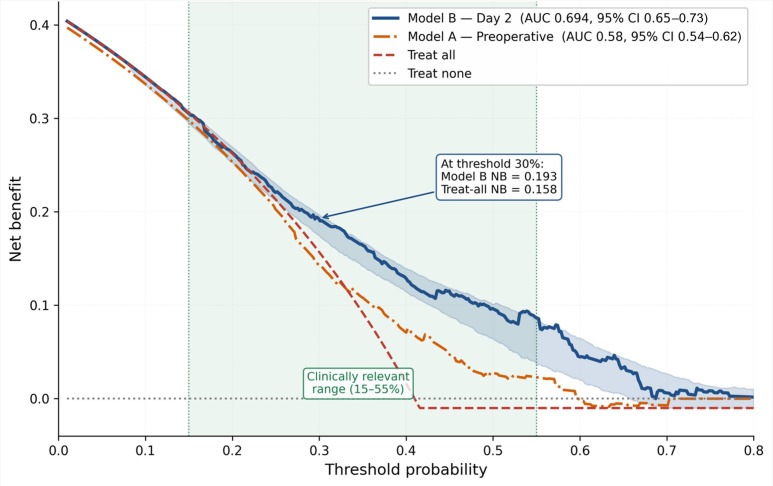
Decision curve analysis (DCA). net clinical benefit of Model B (augmented Day 2; blue, 95% bootstrap CI shaded) and Model A (preoperative; orange dash-dot) compared to ‘Treat all’ (red dashed) and ‘Treat none’ (grey dotted), across threshold probabilities 0–80%. Green shaded area (15–55%) = clinically relevant range. At a 30% threshold, Model B NB = 0.192 vs. 0.157 for ‘Treat all’. *N* = 593 complete cases; 500 bootstrap iterations.

**Figure 3:**
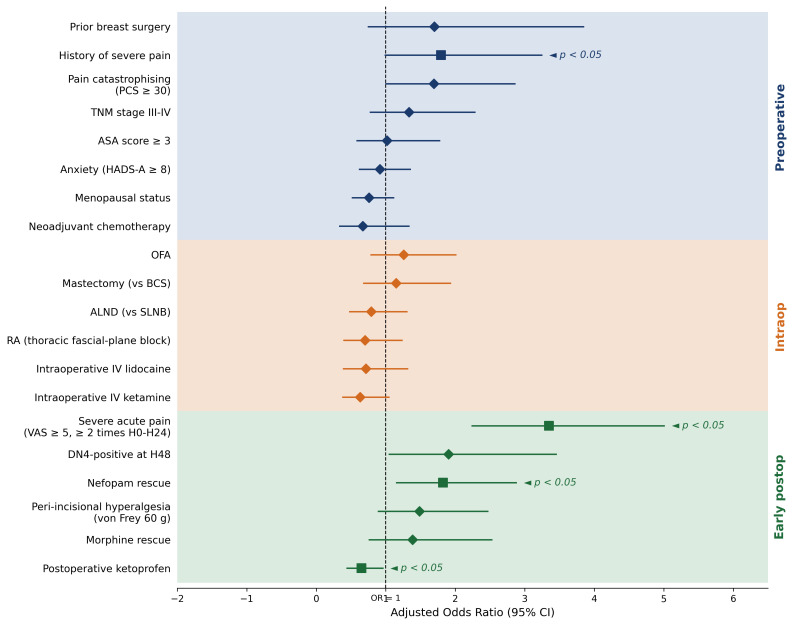
Adjusted odds ratios for all 11 pre-specified variables of Model B. Forest plot (complete cases *N* = 593). Square markers indicate variables significant at *p* < 0.05; diamond markers indicate non-significant variables. Horizontal bars represent 95% confidence intervals. Colours indicate predictor domain: blue = preoperative, orange = intraoperative, green = early postoperative. Protective factors have OR < 1.

Model B (11 pre-specified variables; *N* = 593 complete cases, 243 events, EPV = 22.1) demonstrated markedly superior discrimination (apparent AUC 0.711; optimism-corrected AUC 0.694; 95% CI 0.65–0.73; shrinkage factor 0.93). Although just below the conventional 0.70 threshold, this was accompanied by excellent calibration (intercept −0.01; slope 0.97; Brier score 0.209, a 13.5% improvement over the null-model Brier of 0.242; Hosmer–Lemeshow *p* = 0.42) and net clinical benefit by decision curve analysis across the 15–55% threshold range (Table 4; Figure 2). Receiver Operating Characteristic (ROC) curves comparing Model A and Model B are provided in Figure S2; the LOESS calibration plot for Model B is shown in Figure S3. Regional anaesthesia and intraoperative ketamine did not reach statistical significance in Model B (aOR 0.76 and 0.69, respectively; *p* > 0.10). This is an expected consequence of adjusting for severe acute postoperative pain, which acts as a mediator of their effects (see Section 3.8); this over-adjustment does not invalidate their clinical relevance and is compatible with, though does not independently confirm, an important contribution of severe acute pain prevention to their protective association with CPSP. Five independent predictors reached or approached significance: severe acute postoperative pain (aOR 3.43 [2.40–4.90]; *p* < 0.001), postoperative ketoprofen (aOR 0.66 [0.46–0.96]; *p* = 0.029; protective; E-value 2.40), nefopam rescue (aOR 1.62 [1.05–2.50]; *p* = 0.031), history of severe pain (aOR 1.71 [0.99–2.96]; *p* = 0.044), and pain catastrophising (aOR 1.59 [0.96–2.62]; *p* = 0.069, trend). The full adjusted coefficients for all 11 pre-specified variables are presented in Table 3 and displayed as a forest plot in Figure 3.

### 3.8. Counterfactual Mediation Analysis

For RA, the total effect on CPSP (OR 0.65 [0.44–0.97]) decomposed into a non-significant natural direct effect (NDE OR 0.82 [0.53–1.27]) and a significant indirect effect via severe acute pain (NIE OR 0.79 [0.69–0.90]), corresponding to a mediated proportion of 58.2% (95% CI 41.3–79.6%). For ketamine, the total effect (OR 0.60 [0.42–0.86]) decomposed into NDE (OR 0.80 [0.55–1.17]) and NIE (OR 0.75 [0.66–0.86]), with a mediated proportion of 53.7% (38.2–72.4%). The loss of significance of RA and ketamine in Model B, when fully adjusted for severe acute pain, results from this over-adjustment on a mediator, a feature expected under the mediation framework [27]. E-values for the natural direct effect (RA 1.7; ketamine 1.8) indicate that an unmeasured confounder would need to be associated with both exposure and outcome with an OR of at least 1.7–1.8 to fully explain away the direct effect. The corresponding E-values for the natural indirect effect, the mediation pathway itself, were somewhat larger (RA 1.85; ketamine 2.00), but rest on the additional assumption of no unmeasured mediator–outcome confounding. Given the observational design, these mediation estimates are best interpreted as hypothesis-generating, pending randomised confirmation. Mediated proportions on the risk-difference scale were similar, at 52.1% (RA) and 49.8% (ketamine), reassuring given the high prevalence of the outcome (39.1%). Data is in Table 5. A path diagram of the counterfactual mediation model and a decomposition chart of direct and indirect effects are provided in Figure S4.

### 3.9. Sensitivity Analyses

The dominant predictors of Model B (severe acute pain, ketoprofen, and history of severe pain) remained significant or showed a trend across all eight pre-specified sensitivity analyses (Table S3). The tipping-point analysis shows that the inferences for severe acute pain remain robust unless the OR of missingness is implausibly large (δ > 2.5 for a well-supported predictor), while the smaller effects of history of severe pain and ketoprofen would require δ > 1.5 to be nullified—a threshold that cannot be excluded but is judged unlikely given the MAR mechanism supported by Little’s test. Concordance of log-ORs between complete cases and imputed data: r = 0.97. The LASSO model (R package glmnet, λ_1SE = 0.034; apparent AUC 0.715; 10-fold cross-validated AUC 0.698) retained the same primary predictors with slightly attenuated coefficients due to penalisation (prediction correlation r = 0.96; Table S4). Figure 2 illustrates the decision curve analysis of the final predictive model: the model provided greater net benefit than both the ‘treat all’ and ‘treat none’ strategies across the clinically relevant threshold probability range of 15–55%, supporting its utility for risk stratification in the early postoperative setting. Here, the threshold probability denotes the predicted CPSP risk at or above which clinician and patient would judge a low-burden preventive measure (enhanced multimodal analgesia, psychological support, or closer follow-up) worthwhile; the 15–55% range therefore spans clinically plausible decision thresholds for such interventions.

## 4. Discussion

### 4.1. Relevance and Robustness of Findings

This prospective cohort of 862 analysed patients (out of 1014 enrolled) at the INO Rabat makes three principal contributions. First, it establishes a 3-month CPSP incidence of 39.1% using a strict IASP/IMMPACT composite definition—more demanding than the sole pain intensity criterion still prevalent in the literature—thereby strengthening the clinical significance of identified cases. Second, it provides two complementary, internally validated prediction models usable at two distinct clinical decision points. Third, counterfactual mediation analysis quantifies the proportion of the protective associations of RA and ketamine with CPSP that, under the assumptions of the counterfactual framework, is consistent with mediation through prevention of severe acute pain.

Robustness is supported by consecutive prospective recruitment, strict pre-specification of analyses in a plan signed before the first inclusion (SAP v1.0, December 2020), internal validation by 1000 bootstrap iterations with optimism correction, consistency across eight pre-specified sensitivity analyses, and near-perfect concordance of log-ORs between complete cases and imputed data (r = 0.97). Model B discrimination (optimism-corrected AUC 0.694) is adequate for a clinical prediction model in this domain and is accompanied by good calibration (intercept −0.01; slope 0.97) and a 13.5% improvement in the Brier score relative to the null model.

### 4.2. Predictor Mechanisms

Severe acute postoperative pain (aOR 3.43 [2.40–4.90]) is the strongest predictor in Model B and the central hub of pain chronification: sustained intense nociceptive input induces glutamatergic long-term potentiation in dorsal horn neurons, driving central sensitisation and the transition to chronic pain [9]. Its role as the dominant mediator between perioperative interventions and the 3-month outcome makes it simultaneously a predictor and a primary therapeutic target.

Preoperative pain catastrophising (aOR 1.59 [0.96–2.62]; trend, *p* = 0.069) is accessible at the preanaesthetic consultation via the PCS and is potentially modifiable through targeted preoperative psychological preparation [30]. Its role in amplifying the nociceptive signal via hypervigilance, rumination, and magnification is well established [17].

History of severe pain (aOR 1.71 [0.99–2.96]; *p* = 0.044) signals pre-existing central sensitisation as a vulnerability substrate [10,11], underscoring the importance of structured pain history taking at the preanaesthetic consultation.

Postoperative ketoprofen (aOR 0.66 [0.46–0.96]; *p* = 0.029) constitutes an original finding: its protective effect is independent of severe acute pain in the fully adjusted model, a pattern compatible with a spinal Cyclooxygenase-2 (COX-2) inhibition mechanism beyond simple control of pain intensity [9]; however, as an observational finding subject to potential confounding by indication, it should be regarded as hypothesis-generating. To quantify its robustness, the adjusted ketoprofen effect (aOR 0.66) corresponds to an E-value of 2.40 (E-value for the confidence limit closest to the null: 1.25). Allocation was governed primarily by drug availability and standard contraindications rather than by perceived CPSP risk, which limits but does not eliminate this confounding; the finding therefore supports—but cannot by itself establish—administration of ketoprofen from surgical incision and throughout the first 48 postoperative hours in the absence of contraindications, pending confirmation in a randomised trial.

DN4 at H48 (aOR 1.65 [0.93–2.90]; trend) is retained as a pre-specified early neuropathic marker, reflecting an already-established neuropathic trajectory of the intercostobrachial pathways rather than an independent causal factor, justifying early initiation of neuropathic pain treatment [31]. Nefopam rescue (aOR 1.62 [1.05–2.50]; *p* = 0.031) identifies a distinct analgesic vulnerability phenotype independent of VAS-measured acute pain intensity, making it a simple bedside marker available from the PACU onwards.

RA and ketamine lost statistical significance in Model B once fully adjusted for severe acute pain. This over-adjustment on a mediator is expected under the mediation framework and is compatible with the estimated mediated proportions, but does not constitute independent confirmation, as both analyses draw on the same underlying association. This does not diminish the clinical relevance of RA and ketamine; it is compatible with an important, though not formally proven, contribution of severe acute pain prevention to their protective association with CPSP.

### 4.3. Comparison with the Literature

The incidence of 39.1% lies in the upper range of international series (25–60%) [5,6] and is consistent with regional data from Turkey, where Alkan et al. reported a prevalence of 45.1% across 614 breast cancer survivors in a multicentre study [13], and Beyaz et al. found a prevalence of 64.1% in a cross-sectional cohort of 146 patients [14]. The median age of 48 years is lower than Western medians (60+ years), a profile generally associated with higher CPSP risk, which should be considered when generalising the model to older populations.

The dominant predictors converge with the models of Habib [11] and Schreiber [12]. Our optimism-corrected AUC of 0.694 is slightly below the pooled AUC of 0.73 reported by Wang et al. [32], though our study employs a more rigorous internal validation framework. The independent protective effect of ketoprofen and the identification of nefopam rescue as an analgesic vulnerability marker constitute, to our knowledge, original findings not reported in published prediction models for this population.

The mediated proportions of 58% (RA) and 54% (ketamine) are consistent with what meta-analyses had implicitly suggested [15,16,33]: that these interventions may reduce pain chronification largely through prevention of severe acute pain, under the assumptions of the mediation framework. These proportions are stable across OR-scale and risk-difference-scale analyses (52% and 50%, respectively), reinforcing confidence in the estimates despite the high outcome prevalence.

### 4.4. Limitations

(i) Single-centre design limits external generalisability; multicentre regional validation is ongoing and represents an essential step before formal therapeutic recommendations. (ii) Missing data for some covariates (up to 12.3% for history of severe pain); although the MAR mechanism is formally supported and the tipping-point analysis demonstrates robustness for δ ≤ 1.5–1.8, a Missing Not At Random (MNAR) mechanism cannot be formally excluded. (iii) The three-month follow-up does not capture the effect of adjuvant treatments (radiotherapy and hormone therapy) or the longer-term trajectory at 6–12 months. In our setting, adjuvant radiotherapy is initiated after a median interval of approximately three months from surgery [34]; consequently, no patient had received radiotherapy by the time of the 3-month CPSP assessment, so radiotherapy could not act as a confounder of the present results, and its contribution—together with that of endocrine therapy—can only be assessed in the planned 6- and 12-month follow-up. (iv) Observational mediation analysis: E-values of 1.7–1.8 quantify robustness to unmeasured confounding, but residual confounding by anaesthetist preferences or latent surgical severity cannot be excluded. Additionally, peri-incisional hyperalgesia was assessed by non-calibrated pinprick (Neurotip^®^) rather than von Frey monofilaments; this limits quantitative comparability with studies using standardised mechanical thresholds and may partly explain the non-significant contribution of this variable in the multivariable model. (v) Allocation of RA and ketamine was non-randomised; the resulting confounding was addressed by multivariable modelling and mediation analysis, but cannot be fully eliminated in the absence of randomisation. (vi) The interpretation of nefopam rescue as an analgesic vulnerability marker, rather than merely a proxy for greater pain burden, rests on the residual significance of its aOR after adjustment for VAS-measured acute pain intensity. Although VIF values remained below 2.0 and the correlation between nefopam use and severe acute pain was modest (data in Table S3), residual confounding by unmeasured pain dimensions, particularly movement-evoked or procedural pain, cannot be excluded.

### 4.5. Clinical Implications and Future Directions

Model A (preanaesthetic) enables screening of patients at high psychological risk for routine PCS assessment and RA planning. Model B (Day 2) enables fine-grained risk stratification and targeted resource allocation: early neuropathic pain treatment in DN4-positive patients at H48, intensive psychological support in high-catastrophising patients, and heightened vigilance in patients requiring nefopam rescue. The logistic equation and a worked example are provided in Supplementary S14 (Section S14.3).

The independent protective association of ketoprofen is compatible with its inclusion in early multimodal analgesia protocols at the time of surgical incision, consistent with the institutional protocol already in place and with the mechanistic rationale of spinal COX-2 inhibition limiting central sensitisation; however, as an observational finding, this does not by itself constitute a formal recommendation and requires confirmation in a randomised trial.

Natural extensions include: multicentre regional external validation; 6- and 12-month follow-up incorporating the effect of adjuvant treatments; and a pragmatic randomised trial in high-psychological-risk patients testing a brief preoperative cognitive-behavioural intervention, with mediation analysis formally integrated into the design.

## 5. Conclusions

In this prospective cohort of 862 analysed patients (out of 1014 enrolled) at the Institut National d’Oncologie of Rabat, 39% developed CPSP at 3 months using a strict composite definition. Two complementary, internally validated prediction models, following TRIPOD standards, enable risk stratification at two clinical decision points: Model A (preanaesthetic, AUC 0.58) showed only limited discrimination and is best regarded as an exploratory tool that may flag psychologically at-risk patients for Pain Catastrophising Scale assessment, rather than a stand-alone screening instrument; Model B (postoperative day 2, AUC 0.694) guides targeted prevention. The protective associations of regional anaesthesia and intraoperative ketamine with CPSP were consistent with mediation largely through prevention of severe acute postoperative pain, under the assumptions of the counterfactual framework. Ketoprofen administered from the surgical incision onwards and maintained for 48 h was independently associated with a lower risk of pain chronification; this finding is hypothesis-generating and supports consideration of its inclusion in multimodal analgesia protocols pending confirmation in randomised studies, when available and in the absence of contraindications. To our knowledge, among the first from the MENA region to meet STROBE/TRIPOD standards, these single-centre findings require external multicentre validation across the region before they can be generalised.

## Figures and Tables

**Table 3:** Multivariable logistic regression—Model B (11 pre-specified variables, complete cases *N* = 593, events = 243, EPV = 22.1).

Variable	No CPSP	CPSP	Unadj. OR [95% CI]	Adj. OR [95% CI]	Adj. *p*	VIF	
**Preoperative variables**							
Pain catastrophising (PCS ≥ 30)	12.60%	18.40%	1.56 [1.06–2.30]	1.59 [0.96–2.62]	0.069	1.05	†
History of severe pain	10.80%	15.60%	1.52 [0.99–2.33]	1.71 [0.99–2.96]	0.044	1.07	*
Prior breast surgery	4.10%	6.20%	1.53 [0.82–2.88]	1.82 [0.82–4.05]	0.141	1.03	
Anxiety (HADS-A ≥ 8)	37.70%	37.30%	0.98 [0.73–1.32]	0.94 [0.65–1.36]	0.741	1.04	
ASA score ≥ 3	12.20%	13.10%	1.08 [0.72–1.63]	0.96 [0.57–1.61]	0.881	1.03	
Menopausal status (peri/post vs. pre)	52.00%	48.40%	0.86 [0.66–1.14]	0.85 [0.60–1.22]	0.387	1.03	
TNM stage III–IV	23.10%	27.00%	1.24 [0.90–1.69]	1.36 [0.80–2.31]	0.251	1.79	
Neoadjuvant chemotherapy	12.00%	13.40%	1.13 [0.75–1.70]	0.69 [0.35–1.36]	0.29	1.77	
**Intraoperative variables**							
Mastectomy (vs. BCS)	67.20%	71.50%	1.22 [0.91–1.65]	1.17 [0.72–1.92]	0.522	1.63	
Axillary lymph node dissection	65.30%	69.10%	1.19 [0.89–1.59]	0.77 [0.48–1.23]	0.269	1.53	
RA (thoracic fascial-plane block)	17.10%	11.90%	0.65 [0.44–0.97]	0.76 [0.45–1.29]	0.31	1.05	
Intraoperative IV ketamine	22.90%	15.10%	0.6 [0.42–0.86]	0.69 [0.43–1.11]	0.122	1.07	
Intraoperative IV lidocaine	11.80%	10.70%	0.89 [0.58–1.38]	0.69 [0.38–1.25]	0.221	1.08	
OFA (opioid-free anaesthesia)	19.20%	22.00%	1.18 [0.84–1.65]	1.18 [0.76–1.84]	0.454	1.04	
**Early postoperative variables (H0–H48)**							
Severe acute pain (VAS ≥ 5, ≥ 2 pts H0–H24) ††	30.10%	59.90%	3.48 [2.61–4.63]	3.43 [2.40–4.90]	< 0.001	1.13	***
DN4-positive at H48 (early neuropathic marker)	7.70%	14.50%	2.04 [1.28–3.24]	1.65 [0.93–2.90]	0.084	1.06	†
Peri-incisional hyperalgesia (pinprick, Neurotip^®^)	15.00%	20.60%	1.47 [1.02–2.13]	1.58 [0.98–2.54]	0.059	1.09	†
Postoperative ketoprofen (H0–H48) ‡	54.70%	43.90%	0.65 [0.49–0.86]	0.66 [0.46–0.96]	0.029	1.06	*
Nefopam rescue	23.00%	27.00%	1.24 [0.90–1.69]	1.62 [1.05–2.50]	0.031	1.14	*
Morphine rescue	10.90%	12.50%	1.17 [0.76–1.79]	1.19 [0.67–2.09]	0.552	1.09	

*** *p* < 0.001; * *p* < 0.05; † *p* < 0.10 (trend). Adjusted OR from multivariable logistic regression on complete cases (*N* = 593). All VIF (Variance Inflation Factor) < 2; VIF quantifies multicollinearity: VIF = 1 = no collinearity, >4 requires attention. No significant non-linearity by fractional polynomials. †† Severe acute pain defined as VAS ≥ 5/10 on ≥2 consecutive time points within H0–H24 (not H0–H48), preserving the temporal order for mediation analysis. ‡ Ketoprofen’s protective effect is independent of severe acute pain in the fully adjusted model, consistent with a spinal COX-2 inhibition mechanism. Abbreviations: Adj. OR, adjusted odds ratio; ASA, American Society of Anesthesiologists; BCS, breast-conserving surgery; CI, confidence interval; COX-2, cyclooxygenase-2; DN4, Douleur Neuropathique en 4 questions; EPV, events per variable; HADS-A, Hospital Anxiety and Depression Scale–anxiety subscale; OFA, opioid-free anaesthesia; OR, odds ratio; PCS, Pain Catastrophising Scale; RA, regional anaesthesia; TNM, tumour–node–metastasis; VAS, visual analogue scale; VIF, Variance Inflation Factor; H, hours postoperatively.

**Table 4:** Comparative performance of the two prediction models.

Metric	Model A (Preoperative)	Model B (Augmented, Day 2)	Ideal Value
Events per variable (EPV)	48.1 (7 vars)	22.1 (11 vars)	≥10–20
Apparent AUC	0.581	0.711	≥0.70
Optimism-corrected AUC [95% CI]	0.58 [0.54–0.62]	0.694 [0.65–0.73]	≥0.70
Brier score	0.239	0.209	<0.238 (null)
Improvement vs. null model	−1.9%	−13.5%	↓ better
Calibration intercept	−0.02	−0.01	=0
Calibration slope	0.94	0.97	=1
Hosmer-Lemeshow *p*	0.38	0.42	>0.05
McFadden pseudo-R^2^	0.019	0.089	↑ better
DCA—relevant threshold	Marginal benefit (25–40%)	Net benefit > treat-all and treat-none (15–55%)	Net benefit > 0
Clinical use	Preanaesthetic consultation	Postoperative Day 2 evaluation	—

↓ indicates the percentage reduction in Brier score relative to the null model; a larger negative value reflects greater improvement in model calibration. ↑ indicates that higher values denote better performance; for McFadden’s pseudo-R^2^, larger values reflect greater explanatory power relative to the null model.

**Table 5:** Counterfactual mediation analysis. Proportion of effect mediated through severe acute pain (H0–H24).

Exposure	Total Effect OR [95% CI]	NDE OR [95% CI]	NIE OR [95% CI]	Mediated % [95% CI]	E-Value (NDE)	E-Value (NIE)
RA (thoracic fascial-plane block)	0.65 [0.44–0.97]	0.82 [0.53–1.27]	0.79 [0.69–0.90]	58.2% [41.3–79.6]	1.7	1.85
Intraoperative IV ketamine	0.60 [0.42–0.86]	0.80 [0.55–1.17]	0.75 [0.66–0.86]	53.7% [38.2–72.4]	1.8	2

VanderWeele counterfactual framework; R package mediation v4.5; 1000 bootstrap iterations. Mediator: severe acute pain (H0–H24). Adjusted confounders: age, BMI, TNM stage, surgery type, catastrophising, HADS-D, and ICBN preservation. Exposure × mediator interaction non-significant (*p* > 0.30 for both). E-value (NDE): minimum odds ratio with which an unmeasured confounder, associated with both exposure and outcome, could fully explain away the natural direct effect. E-value (NIE): corresponding value for the natural indirect effect, which additionally assumes no unmeasured mediator–outcome confounding; both computed with the R package EValue on the odds-ratio scale. NDE: natural direct effect; NIE: natural indirect effect; RA: regional anaesthesia.

## Data Availability

De-identified individual participant data and R analysis code are available from the corresponding author (A.G.) upon reasonable request, subject to ethics committee approval (Comité d’Éthique pour la Recherche Biomédicale, Faculté de Médecine et de Pharmacie de Rabat, reference C52-20). The statistical analysis plan (pre-specified, version 1.0, December 2020) is also available upon request.

## Supplementary Materials

The following supporting information can be downloaded at https://www.mdpi.com/article/10.3390/cancers18152433/s1. Table S1: A priori clinical and mechanistic justification of the 11 pre-specified candidate variables (Model B). Table S2: Comparison of baseline characteristics between complete and incomplete observations (standardised differences). Table S3: Summary of eight pre-specified sensitivity analyses. Table S4: LASSO alternative model coefficients (λ_1SE = 0.034). Table S5. Institutional regional anaesthesia allocation algorithm by surgical indication. Figure S1: Missing data distribution (bar chart and heatmap by patient). Figure S2: ROC curves (Model A vs. Model B). Figure S3: Calibration plot(Model B (LOESS)). Figure S4: Counterfactual mediation analysis (path diagram and effect decomposition). Figure S5: Postoperative VAS pain trajectories by CPSP status. Figure S6: CPSP incidence by key predictor strata (univariable analysis). Ref. [35] is cited in Supplementary Materials.

## Abbreviations

The following abbreviations are used in this manuscript:

ASA	American Society of Anesthesiologists
AUC	Area Under the Curve
BPI	Brief Pain Inventory
CPSP	Chronic Post-Surgical Pain
DCA	Decision Curve Analysis
DN4	Douleur Neuropathique en 4 Questions
EPV	Events Per Variable
HADS-A	Hospital Anxiety and Depression Scale—Sous-Score Anxiété
HADS-D	Hospital Anxiety and Depression Scale—Sous-Score Dépression
IASP	International Association for the Study of Pain
CI	CI: Confidence Interval
IMMPACT	Initiative on Methods, Measurement, and Pain Assessment in Clinical Trials
INO	Institut National d’Oncologie
IRC	Institut de Recherche sur le Cancer
IV	Intravenous
LASSO	Least Absolute Shrinkage and Selection Operator
LOESS	Locally Estimated Scatterplot Smoothing
MAR	Missing At Random
MCAR	Missing Completely At Random
MENA	Middle East and North Africa
MICE	Multiple Imputation by Chained Equations
MNAR	Missing Not At Random
NCT	ClinicalTrials.gov identifier
NDE	Natural Direct Effect
NIE	Natural Indirect Effect
OFA	Opioid-Free Anaesthesia
OR	Odds Ratio
ORa/aOR	Odds Ratio Ajusté
PCS	Pain Catastrophizing Scale
PMM	Predictive Mean Matching
RA	Regional Anaesthesia
ROC	Receiver Operating Characteristic
SAP	Statistical Analysis Plan
SD	Standard Deviation
STROBE	Strengthening the Reporting of Observational Studies in Epidemiology
TNM	Tumour Node Metastasis
TRIPOD	Transparent Reporting of a Multivariable Prediction Model for Individual Prognosis or Diagnosis
VAS	Visual Analogue Scale
VIF	Variance Inflation Factor
