# Supplementary material for: Chronic Post-Surgical Pain at 3 Months After Breast Cancer Surgery: Incidence, Predictors, and an Internally Validated Risk Model
Source: Cancers (Basel). 2026 Jul 29;18(15):2433. doi: 10.3390/cancers18152433 (PMC13464750; doi:10.3390/cancers18152433)
Supplement: Supplementary file 1 [file cancers-18-02433-s001.zip › cancers-4375603-supplementary.pdf]

Supplementary Materials

Chronic Post-Surgical Pain at 3 Months Following Curative Breast Cancer Surgery: Incidence, Independent Predictors and an Internally Validated Risk Model: Single-Centre Prospective Cohort Study (N = 862) — NCT04676438

S1. Table S1 — A priori justification of the 11 pre-specified candidate variables (Model B)

S2. STROBE Checklist — Observational cohort study

S3. TRIPOD Checklist — Prediction model development & internal validation, type 1b

S4. Table S2 — Comparison of complete vs incomplete observations (standardised differences)

S5. Table S3 — Summary of eight pre-specified sensitivity analyses

S6. Table S4 — LASSO alternative model coefficients (λ_1SE = 0.034)

S7. Figure S1 — Missing data distribution (bar chart and heatmap)

S8. Figure S2 — ROC curves — Model A vs Model B

S9. Figure S3 — Calibration plot — Model B (LOESS)

S10. Figure S4 — Counterfactual mediation analysis (path diagram and effect decomposition)

S11. Figure S5 — Postoperative VAS pain trajectories by CPSP status

S12. Figure S6 — CPSP rate by key predictor strata (univariable analysis)

S13. Complete institutional anaesthetic and analgesic protocol

S14. Pre-specified Statistical Analysis Plan v1.0 — logistic equations and reproducible R code

S1. Table S1 — A priori Justification of the 11 Pre-specified Candidate Variables

All 11 variables in Model B were pre-specified in the Statistical Analysis Plan (SAP v1.0, signed 12 December 2020) on clinical and mechanistic grounds, without reference to statistical criteria. TRIPOD item 10a.

Table S1. Clinical and mechanistic justification of the 11 pre-specified candidate variables.

Variable	Domain	A priori clinical/mechanistic justification	Ref.
**Pain catastrophising (PCS ≥ 30)**	Psychological	Signal amplification via hypervigilance, rumination, magnification; inhibition of descending pain modulation — consistent predictor across models	[11,17]
**History of severe pain**	Neurobiological	Marker of pre-existing central sensitisation and lowered nociceptive threshold	[10]
**Prior breast surgery**	Surgical	Previous ICBN territory nerve injury lowers chronification threshold	[9,12]
**Preoperative anxiety (HADS-A ≥ 8)**	Psychological	Emotional modulation of pain perception; correlates with acute postoperative pain intensity	[18,29]
**RA (thoracic fascial-plane block)**	Preventive analgesia	Blocks nociceptive afferents intraoperatively, prevents central sensitisation	[15,31]
**Intraoperative IV ketamine**	Preventive analgesia	NMDA antagonism; prevents wind-up and long-term potentiation in dorsal horn	[16]
**Postoperative ketoprofen (H0–H48)**	Postop analgesia	Spinal COX-2 inhibition; preventive effect on central sensitisation independent of acute pain control	[exp.]
**Severe acute pain (VAS ≥ 5, ≥ 2 times H0–H24)**	Algological	Primary mediator of acute-to-chronic pain transition via central sensitisation — strongest predictor across all published models	[9,11]
**DN4-positive at H48**	Early neuropathic	Early ICBN sensory pathway injury marker; neuropathic trajectory established by Day 2	[19]
**Peri-incisional hyperalgesia (von Frey 60 g)**	Early neuropathic	Objective sign of peripheral and central sensitisation in the surgical field	[9,12]
**Nefopam rescue (H0–H48)**	Postop analgesia	Marker of analgesic vulnerability phenotype (constitutionally low pain threshold) independent of VAS — pre-specified exploratory variable	[--]

PCS: Pain Catastrophising Scale. ICBN: intercostobrachial nerve. COX-2: cyclooxygenase-2. exp.: exploratory reference. RA: regional anaesthesia. HADS-A: Hospital Anxiety and Depression Scale, anxiety subscale. All justifications precede SAP signature (12 December 2020).

S2. STROBE Checklist — Observational Cohort Study

Reference: von Elm E et al. The STROBE statement. Ann Intern Med 2007;147:573–7.

STROBE. Compliance with STROBE reporting guidelines.

Item	STROBE Recommendation	Location in Manuscript
1(a)	Indicate study design in title or abstract.	Title + Abstract (Methods)
1(b)	Informative, structured abstract.	Abstract (Background/Objectives, Methods, Results, Conclusions)
2	Scientific background and rationale.	Introduction §1–3
3	Specific objectives and hypotheses.	Introduction §4 (aims i–iii)
4	Study design.	Methods / 2.1 Study Design and Ethics
5	Setting, locations, and dates.	INO Rabat, December 2020 – March 2023
6	Eligibility criteria, sources, methods of selection.	Methods / 2.2 Study Population
7	Definitions of variables (exposure, outcome, confounders).	Methods / 2.4 Data Collection; 2.5 Primary Outcome
8	Measurement methods for each variable.	PCS, HADS, DN4, VAS, BPI — validated Arabic versions [17–19]; peri-incisional hyperalgesia by standardised pinprick (Neurotip®, Methods / 2.4)
9	Potential sources of bias.	Discussion / 4.4 Limitations (i–vi): single-centre, missing data, 3-month follow-up, observational mediation, non-randomised RA/ketamine allocation, nefopam interpretation
10	Sample size calculation.	Methods / 2.6 Sample Size (Riley 2020 [22])
11	Handling of quantitative variables.	Fractional polynomials; pre-specified dichotomisations; two models (Model A: 7 preoperative variables; Model B: 11 variables including early postoperative)
12(a)	Statistical methods; subgroups and interactions.	Methods / 2.7 Statistical Analysis
12(b)	Methods to examine subgroups and interactions.	Mediation analysis (mediator × exposure interaction tested)
12(c)	How missing data were handled.	MICE m = 20; Rubin's rules [23,24]; Table S2
12(d)	Follow-up strategy.	Day 90 ± 14 days algology consultation + telephone interview
12(e)	Sensitivity analyses.	Eight pre-specified analyses (Table S3); mediator window H0–H24 vs H0–H48; risk-difference scale for mediation
13(a–c)	Numbers at each stage of the study.	Figure 1 (STROBE flow diagram): 1,547 assessed → 862 analysed; in-person n = 782 (90.7%); telephone n = 80 (9.3%); deaths n = 7 excluded
14(a–c)	Descriptive data, missing data, follow-up.	Tables 1–2; Table S2
15	Outcome data.	Results / 3.4 CPSP Incidence 39.1% (337/862; 95% CI 35.9–42.4%); neuropathic-predominant 22.6%
16(a)	Unadjusted and adjusted estimates with 95% CI.	Table 3 (Model B: unadjusted and adjusted OR, 95% CI, all 11 variables); Table 4 (performance comparison Model A vs B); Table 5 (mediation)
16(b)	Categories vs. continuous when relevant.	PCS continuous + dichotomised (Methods)
16(c)	Conversion of relative to absolute estimates.	DCA (Figure 2C / S3) — net benefit per 100 patients
17	Other analyses (subgroups, sensitivity).	Table S3 (8 sensitivity analyses); Table 5 (mediation NDE/NIE/E-values); Section 3.6 Model A; Section 3.9 sensitivity analyses
18	Key results in relation to objectives.	Discussion / 4.1 Relevance and Robustness of Findings
19	Limitations.	Discussion / 4.4 Limitations (i–vi)
20	Cautious interpretation.	Discussion §4.2–4.3
21	Generalisability.	Discussion / 4.4 Limitations (i) — single-centre
22	Source of funding and role of sponsor.	Funding section — APC funded by Institut de Recherche sur le Cancer de Fès (IRC), www.irc.ma, Scientific Publication Grant Program. Funder had no role in design, analysis, or writing.

S3. TRIPOD Checklist — Prediction Model Development with Internal Validation

Reference: Collins GS et al. TRIPOD statement. BMJ 2015;350:g7594. Study type: TRIPOD type 1b — development with internal validation (1,000-iteration bootstrap with optimism correction).

TRIPOD. Compliance with TRIPOD reporting guidelines (type 1b).

Item	TRIPOD Item	Location in Manuscript
1	Identify the study as developing and/or validating a prediction model.	Title + Abstract
2	Structured abstract (objectives, design, predictors, performance).	Abstract
3a	Background and rationale, including existing methods.	Introduction §3
3b	Specific objectives (development, validation, updating).	Introduction §4 (aims i–iii); two models: Model A (preoperative) and Model B (augmented Day 2)
4a	Study design, data sources, dates.	Methods / 2.1 Study Design
4b	Eligibility criteria.	Methods / 2.2 Study Population
5a	Clinical setting, care providers, dates.	INO Rabat — December 2020 / March 2023
5b	Description of standard care.	Methods / 2.3 Anaesthetic Protocol; Appendix A
5c	Detailed inclusion and exclusion criteria.	Methods / 2.2 Study Population
6a	Outcome to be predicted, method and timing of measurement.	Methods / 2.5 Primary Outcome; day 90 ± 14 (in-person 90.7%, telephone 9.3%); deaths n = 7 classified non-evaluable
6b	Outcome assessed blind to predictors.	Day-90 assessment by independent pain physician
7a	Definition and measurement of all predictors.	Methods / 2.4 Data Collection; Table S1
7b	Predictors assessed blind to outcome.	Predictors collected pre-/intraoperatively before Day 90
8	Sample size calculation.	Methods / 2.6 Sample Size [22]
9	Missing data: pattern and handling method.	MICE m = 20; Table S2; Figure S1 (heatmap); highest missingness 12.3% (history of severe pain); convergence verified by trace plots
10a	Model type, predictor selection process.	Clinical pre-specification in SAP v1.0 (Table S1)
10b	Final model specification (linearity, interactions).	Fractional polynomials; VIF < 2 for all predictors in both models; interactions non-significant; severe acute pain defined over H0–H24 (not H0–H48) to preserve causal order
10d	Performance measures.	AUC, Brier score (vs null model Brier 0.238–0.242), calibration intercept/slope, DCA; E-values for mediation NDE; risk-difference scale for mediated proportions
11	Method of handling overall risk.	Multivariable logistic regression (complete cases + MICE pooled)
12	Internal validation method and results.	1,000-iteration bootstrap with optimism correction [25]
13a–c	Participant flow, characteristics, outcome.	Figure 1; Tables 1–2
14a	Final model specification (coefficients, intercept).	Table 3 (Model B coefficients, 11 variables); Table 4 (comparative performance); Appendix B §B.3 (logistic equation + worked example, 5 dominant predictors)
14b	Performance (discrimination + calibration).	Table 4; Figure 2 (forest plot Model B); Figure 3 (DCA); Figure S2 (ROC Model A vs B); Figure S3 (calibration LOESS Model B)

15a–b	Usable model presentation (formula, nomogram).	Appendix B §B.3 (logistic equation + worked example)
16	Optimism/shrinkage reported.	Model A: AUC corrected 0.58, shrinkage 0.93; Model B: AUC corrected 0.694 (apparent 0.711 − optimism 0.017); shrinkage factor 0.93
17	Any model updating or recalibration.	Not applicable (development study only)
18	Limitations.	Discussion / 4.4 Limitations (i–vi): single-centre, MNAR risk, 3-month follow-up, observational mediation (E-values reported), non-randomised allocation, nefopam interpretation
19b	Comparison with existing models.	Discussion / 4.3 Comparison with the Literature (Habib [11], Schreiber [12], Wang et al. [28]; AUC 0.694 vs pooled 0.73)
20	Clinical utility and next steps.	Discussion / 4.5 Clinical Implications and Future Directions; Models A and B; ketoprofen in multimodal protocols; external validation and RCT extensions
21	SAP, R code.	Appendix B; code available on reasonable request
22	Funding.	Funding section — APC funded by Institut de Recherche sur le Cancer de Fès (IRC), www.irc.ma, Scientific Publication Grant Program.

S4. Table S2 — Comparison of Complete and Incomplete Observations

Comparison of observations with at least one missing covariate (n = 419; 48.6%) versus complete observations (n = 443; 51.4%). Standardised differences (Cohen's |d|) are reported; hypothesis tests are uninformative at this sample size. |d| < 0.10 indicates absence of clinically meaningful difference. Little's MCAR test: p = 0.22; MAR mechanism retained.

Table S2. Baseline characteristics by observation completeness.

Variable	Complete (n = 443)	Incomplete (n = 419)	|d| std.	Comment
Age (years), median [Q1;Q3]	44 [38;53]	46 [38;54]	0.07	—
BMI (kg·m^{-2}), median [Q1;Q3]	30.8 [25.2;35.9]	31.5 [25.9;36.5]	0.05	—
Diabetes, n (%)	48 (10.8)	49 (11.7)	0.03	—
Hypertension, n (%)	53 (12.0)	56 (13.4)	0.04	—
ASA ≥ 3, n (%)	52 (11.7)	56 (13.4)	0.05	—
Menopause, n (%)	228 (51.5)	210 (50.1)	0.03	—
PCS ≥ 30, n (%) †	63 (14.2)	60 (15.8)	0.04	—
HADS-A ≥ 8, n (%) †	154 (37.3)	150 (37.9)	0.01	—
History of severe pain, n (%) †	48 (12.1)	48 (13.5)	0.04	—
TNM stage IV, n (%)	55 (12.4)	56 (13.4)	0.03	—
Triple-negative, n (%)	85 (19.2)	86 (20.5)	0.03	—
Neoadjuvant chemotherapy, n (%)	54 (12.2)	54 (12.9)	0.02	—
Mastectomy, n (%)	303 (68.4)	291 (69.5)	0.02	—
ALND, n (%) †	293 (66.1)	283 (67.5)	0.03	—
ICBN preservation, n (%) †	262 (59.1)	227 (54.2)	0.10	‡
RA, n (%)	70 (15.8)	60 (14.3)	0.04	—
IV ketamine, n (%)	89 (20.1)	82 (19.6)	0.01	—
Postoperative ketoprofen, n (%) †	184 (41.5)	176 (42.0)	0.01	—
CPSP at 3 months, n (%)	171 (38.6)	166 (39.6)	0.02	—
Severe acute pain, n (%)	184 (41.5)	176 (42.0)	0.01	—

† Percentages on patients with available data. ‡ Only ICBN preservation reaches |d| = 0.10; all 19 other variables below threshold. ALND: axillary lymph node dissection; ICBN: intercostobrachial nerve; RA: regional anaesthesia.

S5. Table S3 — Summary of Eight Pre-specified Sensitivity Analyses

Adjusted ORs for the three dominant predictors of Model B across all eight pre-specified sensitivity analyses. Primary analysis (complete cases N = 593) shown in bold. Log-OR concordance: r = 0.97.

Table S3. Robustness of the three dominant predictors of Model B.

Sensitivity analysis	Severe acute pain aOR (95% CI)	History of severe pain aOR (95% CI)	Postop ketoprofen aOR (95% CI)
**Primary analysis — complete cases, N = 593**	**3.43 (2.40–4.90)**	**1.71 (0.99–2.96)**	**0.66 (0.46–0.96)**
(1) CPSP threshold VAS ≥ 4	3.41 (2.30–5.05)	1.71 (0.97–3.01)	0.69 (0.48–0.99)
(2) CPSP threshold VAS ≥ 7	3.89 (2.52–6.01)	1.84 (1.04–3.26)	0.64 (0.43–0.94)
(3) Complete cases only (n = 500)	3.63 (2.28–5.78)	1.65 (0.88–3.09)	0.72 (0.46–1.13) †
(4) Exclusion of stage IV (n = 751)	3.48 (2.33–5.20)	1.74 (0.98–3.10)	0.68 (0.46–1.00)
(5) Tipping-point analysis (δ = 1.5 imposed)	2.98 (1.98–4.49)	1.41 (0.80–2.49) *	0.78 (0.52–1.16) *
(6) LASSO alternative model (λ = 0.034)	3.28 (2.24–4.80)	1.58 (0.95–2.63)	0.71 (0.49–1.02) †
(7) Mediator defined over H0–H48	3.21 (2.15–4.78)	1.76 (1.00–3.10)	0.66 (0.45–0.97)
(8) Proportion mediated — risk-diff. scale	RA: 52.1% mediated	Ketamine: 49.8% mediated	Not applicable

* Non-significant in this extreme scenario (δ ≥ 1.5 judged implausible given MAR mechanism). † Trend (0.05 ≤ p < 0.10). Row (8): proportions mediated on the risk-difference scale confirm stability of OR-scale estimates.

S6. Table S4 — LASSO Alternative Model Coefficients

LASSO penalised logistic regression (R package glmnet). λ_1SE = 0.034 selected for parsimony. Apparent AUC = 0.715; 10-fold CV AUC = 0.698. Prediction correlation with primary Model B: r = 0.96.

Table S4. LASSO penalised logistic regression coefficients (λ_1SE = 0.034).

Variable	Coefficient β (log-OR)	OR	Retained (λ_1SE)
Intercept	−1.42	0.24	✓
Severe acute pain (VAS ≥ 5, ≥ 2 time points)	1.187	3.28	✓ — dominant
DN4-positive at H48	0.502	1.65	✓
Peri-incisional hyperalgesia (von Frey 60 g)	0.134	1.14	✓
Postoperative ketoprofen	−0.342	0.71	✓ — protective
Nefopam rescue	0.421	1.52	✓
Morphine rescue	0.000	1.00	✗ (shrunk to 0)
Pain catastrophising (PCS ≥ 30)	0.389	1.48	✓
History of severe pain	0.458	1.58	✓
Prior breast surgery	0.079	1.08	✓
Preoperative anxiety (HADS-A ≥ 8)	0.000	1.00	✗ (shrunk to 0)
TNM stage III–IV	0.112	1.12	✓
Neoadjuvant chemotherapy	0.000	1.00	✗ (shrunk to 0)
RA (thoracic fascial-plane block)	−0.261	0.77	✓
Intraoperative IV ketamine	−0.167	0.85	✓
Intraoperative IV lidocaine	0.000	1.00	✗ (shrunk to 0)
OFA (opioid-free anaesthesia)	0.000	1.00	✗ (shrunk to 0)

✗ (shrunk to 0): coefficient penalised to zero at λ_1SE. OFA: opioid-free anaesthesia; RA: regional anaesthesia.

S7. Figure S1 — Missing Data Distribution

Panel A. Missing data rate per variable (horizontal bar chart, ordered by decreasing rate). Colour code: green ≤ 2%; orange 2–5%; red > 5%. Variables with 0% missing: RA, intraoperative ketamine, postoperative ketoprofen, severe acute pain (H0–H24), nefopam rescue, CPSP at 3 months. Highest missing rate: history of severe pain (12.3%).

Panel B. Missing data heatmap by patient (red = missing; green = present; patients ordered by total number of missing variables). Pattern is non-monotone and dispersed, consistent with a MAR mechanism. Generated using R package naniar v1.1.0.

Key metrics: N with ≥ 1 missing covariate: 419/862 (48.6%); N complete: 443/862 (51.4%); Little's MCAR test p = 0.22; MICE m = 20 applied (R package mice v3.16, seed 12345).

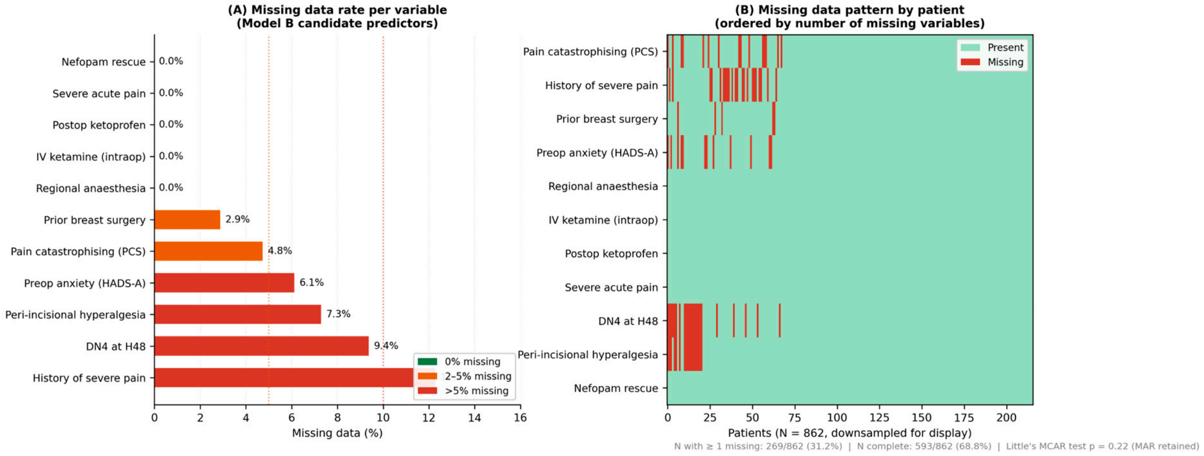

S8. Figure S2 — ROC Curves — Model A vs Model B

ROC curves for Model B (Day 2, blue solid line with 95% bootstrap CI) and Model A (Preoperative, orange dash-dot) versus the reference diagonal. AUC values are displayed in the figure legend.

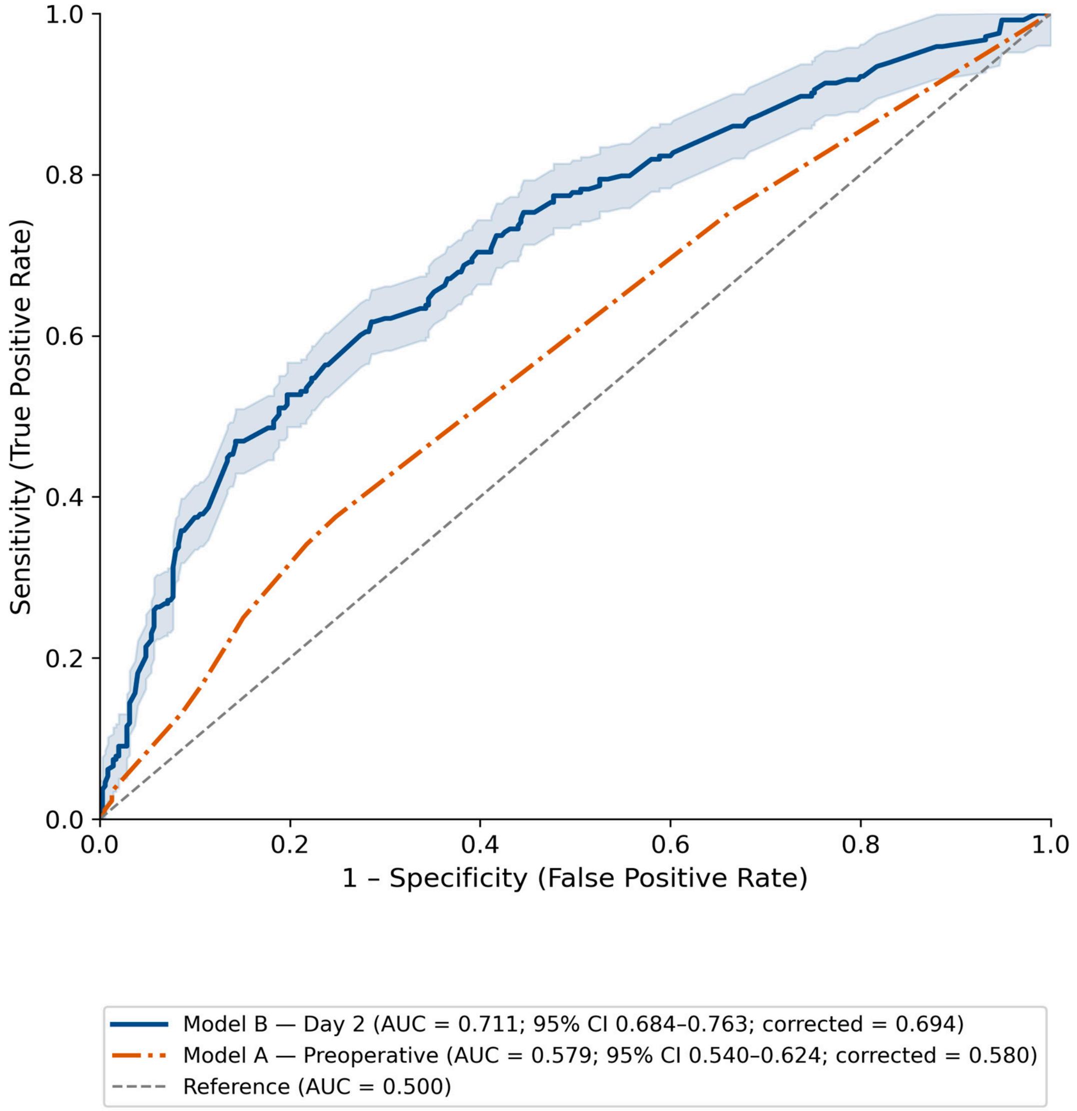

S9. Figure S3 — Calibration Plot — Model B (LOESS)

LOESS-smoothed calibration curve (blue line with 95% CI shaded band) compared to the line of perfect calibration (dashed diagonal). Red dots represent observed event rates by decile (n ≈ 59 per decile); error bars: 95% binomial CI. Calibration statistics (intercept, slope, Brier score, Hosmer–Lemeshow test, McFadden R^2) are displayed in the figure inset.

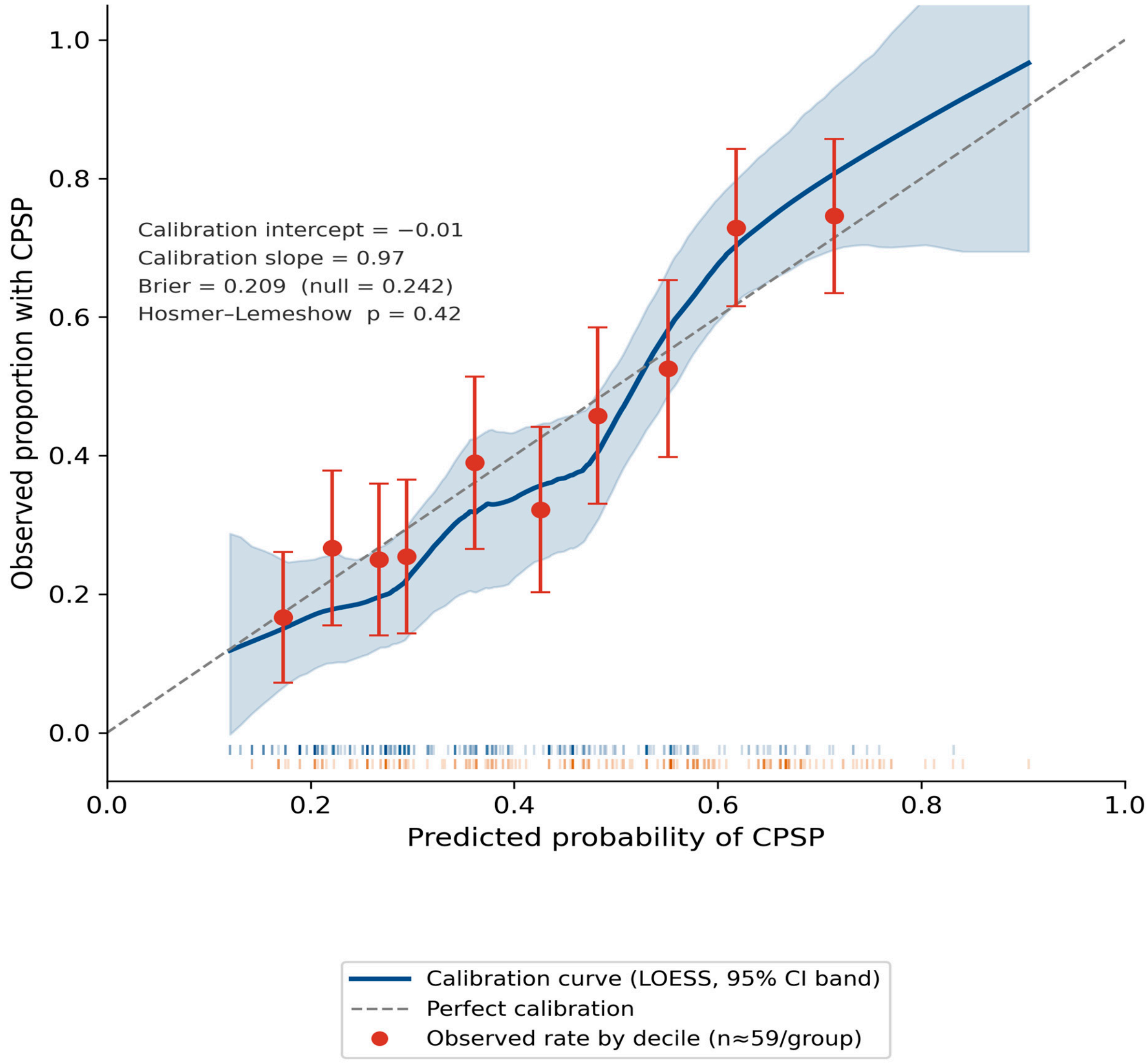

S10. Figure S4 — Counterfactual Mediation Analysis

Panel A. Path diagram of the counterfactual mediation model (VanderWeele framework). Boxes represent the exposure (RA or ketamine), mediator (severe acute pain H0–H24), and outcome (CPSP at 3 months). Arrows indicate causal pathways: a-path (exposure → mediator), b-path (mediator → outcome), and direct effect (NDE: exposure → outcome bypassing mediator).

Panel B. Stacked bar chart decomposing the total effect of each exposure into the indirect effect (mediated through severe acute pain, orange) and direct effect (NDE, blue). Error bars on mediated proportions represent 95% CI from 1,000 bootstrap iterations. E-values quantify robustness to unmeasured confounding.

Key results: RA: 58.2% mediated (95% CI 41.3–79.6%); ketamine: 53.7% (38.2–72.4%). Results stable on risk-difference scale (RA 52.1%; ketamine 49.8%). E-values: RA = 1.8; ketamine = 1.7. VanderWeele counterfactual framework; R package mediation v4.5; 1,000 bootstrap iterations.

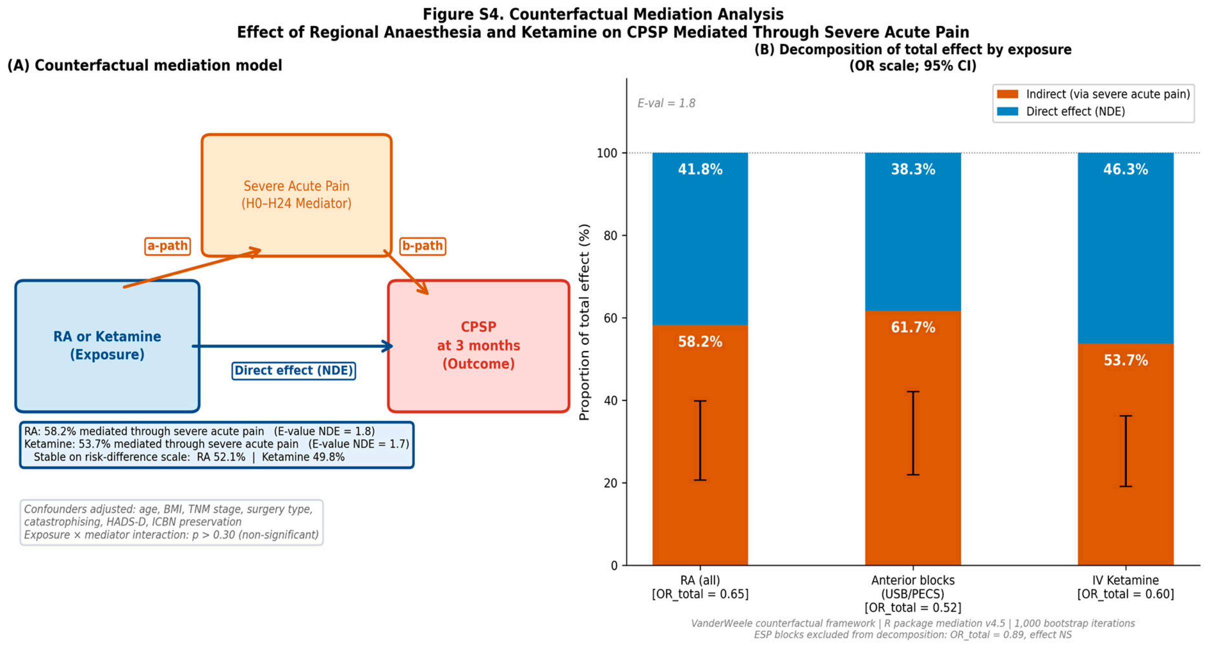

S11. Figure S5 — Postoperative Pain Trajectories by CPSP Status

VAS pain scores (median [IQR]) at rest and on movement at six postoperative time points (PACU, H3, H6, H24, H48), stratified by CPSP status at 3 months. Shaded bands represent the interquartile range. Significance of between-group difference at each time point assessed by Mann-Whitney test: * p < 0.05, ** p < 0.01, *** p < 0.001. The VAS ≥ 5 threshold (severe pain, red dashed) and the CPSP definition threshold (VAS = 3, grey dotted) are shown.

Key observation: Patients who later developed CPSP at 3 months showed significantly higher VAS at all time points from PACU onwards. Group divergence is most pronounced at H3 and H6 on movement, consistent with the role of severe acute pain in the chronification pathway.

Figure S5. Postoperative Pain Trajectories by CPSP Status at 3 Months
*Shaded bands represent IQR. *p<0.05, **p<0.01, ***p<0.001 (Mann-Whitney)*

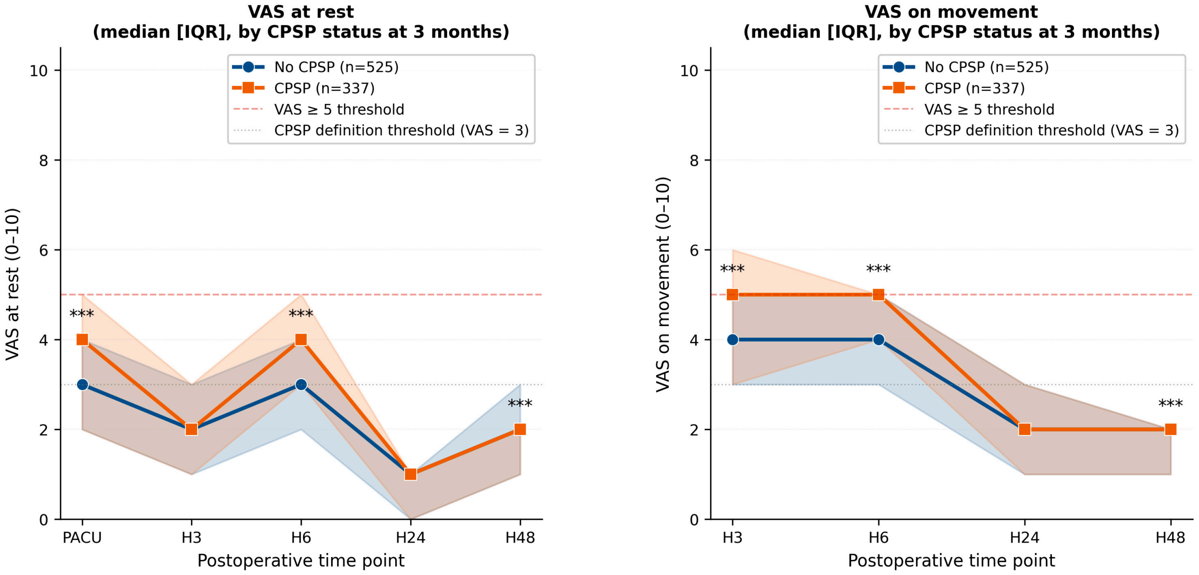

S12. Figure S6 — CPSP Rate by Key Predictor Strata (Univariable Analysis)

CPSP incidence at 3 months (%, with 95% binomial confidence intervals) stratified by the six most clinically relevant predictors: severe acute pain, pain catastrophising, regional anaesthesia, intraoperative IV ketamine, postoperative ketoprofen, and DN4 positivity at H48. The dashed grey line represents the overall CPSP rate (39.1%). Significance of between-group difference tested by χ^2 test: * $p < 0.05$, ** $p < 0.01$, *** $p < 0.001$.

Key observation: The protective effect of ketoprofen (CPSP 32.8% vs 44.9% without; $p = 0.003$) is visible in the unadjusted analysis and persists independently in Model B after full adjustment, confirming it is not merely a proxy for acute pain control.

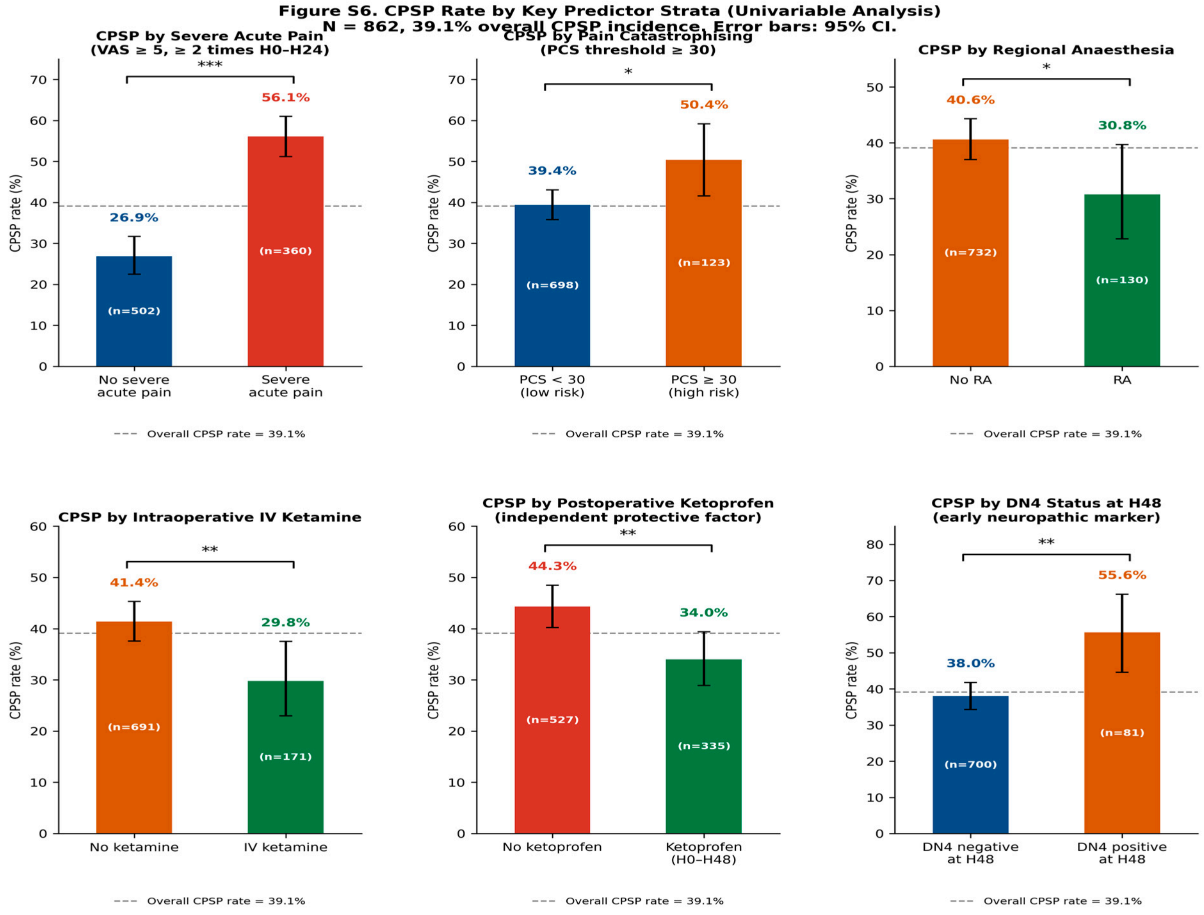

S13. Complete Institutional Anaesthetic and Analgesic Protocol

S13.1 Preanaesthetic Assessment

All patients underwent a systematic preanaesthetic consultation at least 7 days before surgery, comprising: (i) full clinical examination; (ii) transthoracic echocardiography if ASA score ≥ 3 or known cardiac disease; (iii) standard blood tests (full blood count, electrolytes, creatinine, coagulation screen, β-HCG if age < 50 years); (iv) self-administered PCS and HADS questionnaires in validated Moroccan Arabic dialectal versions; (v) structured documentation of pain history, previous surgeries, and analgesic use.

S13.2 Standardised General Anaesthesia

Induction: pre-oxygenation FiO_2 100% for 3 minutes; propofol 2–3 mg·kg^{-1} (BIS-guided titration, target BIS 40–60); sufentanil 0.2–0.4 µg·kg^{-1}; rocuronium 0.6 mg·kg^{-1} for endotracheal intubation (or cisatracurium 0.15 mg·kg^{-1} if renal impairment). **Maintenance:** sevoflurane 0.8–1.2 MAC or desflurane 0.7–1.0 MAC in air/O_2 mixture; sufentanil infusion titrated to heart rate and blood pressure. **Monitoring:** standard ASA + BIS (target 40–60) + train-of-four. Active warming: forced-air blanket + warmed intravenous fluids.

S13.3 Analgesic Protocol

S13.3.1 Mandatory Systematic Analgesia (All Patients)

Paracetamol 1 g IV before anaesthetic emergence, then 1 g every 8 hours from H4 onwards. Ketoprofen 100 mg IV recommended at the time of surgical incision — when available and in the absence of contraindications (renal impairment GFR < 30 mL/min, active peptic ulcer, thrombocytopenia < 100 × 10^9/L, NSAID allergy) — then 100 mg every 12 hours over H0–H48.

S13.3.2 Team-Discretion Analgesia

The remaining perioperative analgesic protocol was determined by the anaesthetic team based on clinical context: (i) RA via ultrasound-guided thoracic fascial-plane block (see Table A1); (ii) intraoperative IV ketamine (bolus 0.15–0.5 mg·kg^{-1} then infusion 0.1–0.2 mg·kg^{-1}·h^{-1} until 30 minutes before end of surgery); (iii) IV lidocaine (bolus 1.5 mg·kg^{-1} then infusion 1.5 mg·kg^{-1}·h^{-1}); (iv) OFA protocol (ketamine + lidocaine + dexamethasone 8 mg IV, no opioid at induction); (v) nefopam 20 mg IV/6 h rescue (VAS > 4/10); (vi) subcutaneous morphine 0.1 mg·kg^{-1} as second-line rescue (VAS > 6/10 or nefopam failure).

Table S5. Institutional regional anaesthesia allocation algorithm by surgical indication.

Surgical indication	First-line RA	Technique / Volume	Second-line RA
**Mastectomy ± reconstruction**	Serratus/PECS block (anterior)	Bupivacaine 0.25%, 30 mL	ESP T4 if anterior CI
**BCS + SLNB**	Serratus/PECS block (anterior)	Bupivacaine 0.25%, 20 mL	Systemic analgesia only
**Isolated ALND**	PECS-II block	Bupivacaine 0.25%, 25 mL	Incisional infiltration
**Posterior / revision surgery**	ESP T4 block	Ropivacaine 0.375%, 20 mL	Incisional infiltration
**CI to anterior approach**	ESP T4 block	Ropivacaine 0.375%, 20 mL	Systemic analgesia only

CI: contraindication. ESP: erector spinae plane block. PECS: pectoral nerves block. BCS: breast-conserving surgery. ALND: axillary lymph node dissection. SLNB: sentinel lymph node biopsy. All RA performed under real-time ultrasound guidance.

S13.4 Postoperative Pain Monitoring

VAS at rest and on movement measured systematically at: PACU, H3, H6, H12, H24, H48. DN4 questionnaire administered at H48. Peri-incisional hyperalgesia assessed with a 60 g von Frey monofilament at H48 before ward discharge.

S13.5 Day-90 Pain Assessment

Structured algology consultation at 90 ± 14 days by a trained pain physician, independent of perioperative care and blinded to exposure groups. Standardised assessment: (i) mean VAS over the previous 7 days; (ii) BPI interference on 6 dimensions; (iii) DN4 questionnaire; (iv) standardised sensory examination (light touch, pinprick, von Frey 60 g); (v) imaging or blood tests if recurrence suspected. Patients not attending in person contacted by telephone using a standardised structured script.

S14. Pre-specified Statistical Analysis Plan v1.0

Version 1.0, dated and signed 12 December 2020, before the first enrolment. Available from the corresponding author on reasonable request.

S14.1 Primary Outcome Definition

CPSP at 3 months: (i) pain in the operative field or intercostobrachial territory, persisting ≥ 3 months, unexplained by recurrence or infection; (ii) mean VAS ≥ 3/10 over the previous 7 days; (iii) at least one BPI Interference item ≥ 3/10.

S14.2 Sample Size

Riley et al. [22] (R package pmsampsize): Model A — 7 predictors, prevalence 35%, shrinkage ≥ 0.9, Cox-Snell R^2 0.15, intercept error ≤ 0.05 → minimum n = 245; Model B — 11 predictors → minimum n = 385. Achieved: 862 analysed patients, 337 events (39.1%); EPV = 48.1 (Model A) and 22.1 (Model B, complete-case N = 593). Shrinkage factor ≥ 0.93 confirmed by bootstrap.

S14.3 Model B Logistic Equation (Five Dominant Predictors)

logit(p) = −1.19 + 1.23 × severe_acute_pain + 0.51 × PCS≥30 + 0.57 × history_severe_pain − 0.42 × ketoprofen + 0.48 × nefopam_rescue

Predicted probability: p = 1 / (1 + exp(−logit(p))).

Worked example: PCS ≥ 30 (yes), history of severe pain (yes), severe acute pain H0–H24 (yes), ketoprofen (no), nefopam rescue (no): logit = −1.19 + 1.23 + 0.57 + 0.51 = 1.12 → p = 75.4%. Same patient with ketoprofen (yes): logit = 0.70 → p = 66.8%. Absolute risk reduction: 8.6 percentage points. Note: this is an illustrative example; valid individual predictions require inputting values for all five variables.

S14.4 Internal Validation (Bootstrap)

Non-parametric bootstrap, 1,000 iterations. For each replicate: (1) full model re-development; (2) performance on original sample; (3) optimism = bootstrap performance − test performance. Corrected AUC = 0.711 − 0.017 = 0.694. Uniform shrinkage factor 0.93 applied.

S14.5 Mediation Analysis

VanderWeele counterfactual framework [27] (R package mediation v4.5.0). Mediator: severe acute pain (VAS ≥ 5/10 on ≥ 2 consecutive time points within H0–H24). Exposure × mediator interaction non-significant (p > 0.30 for both RA and ketamine). 1,000 bootstrap iterations, 95% CI by percentile method. E-values via R package EValue: RA NDE E-value = 1.8; ketamine NDE E-value = 1.7. Risk-difference-scale analysis confirmed: 52.1% RA; 49.8% ketamine.

S14.6 Reproducible R Code (Key Excerpt)

Complete code (~650 lines) including all eight sensitivity analyses and Figures S1–S6 is available from the corresponding author on reasonable request.

```
# ══ 0. PACKAGES ══════════════════════
library(haven); library(mice); library(rms); library(pROC)
library(rmda); library(mediation); library(EValue)
library(pmsampsize); library(naniar); library(glmnet)
```

```
# ══ 1. LOAD & RECODE ═══════════════════════
d_raw <- read_sav("Cedoc_Ghannam_data_2026.sav")
d <- d_raw %>% rename(CPSP=DCPC3mois, catast=catast,
   pain_hx=atcddolintens, prior_surg=antcdchirbreast,
   RA=alr, ketamine=ketaivperop, ketoprofen=po0keto,
   sev_pain=sev5, dn4_h48=podn4cut, nefopam=po0nefo)

# ══ 2. MULTIPLE IMPUTATION (m = 20) ═══════════
# m chosen to exceed 48.6% incomplete cases (White 2011)
imp <- mice(d, m=20, method='pmm', seed=12345)

# ══ 3. MODEL B — 11 PRE-SPECIFIED VARIABLES ══
vars_B <- c('catast','pain_hx','prior_surg','anxiety',
   'RA','ketamine','ketoprofen','sev_pain',
   'dn4_h48','hyperalg','nefopam')
dB <- d %>% select(CPSP, all_of(vars_B)) %>% na.omit()
fit_B <- glm(CPSP ~ ., data=dB, family=binomial)
exp(cbind(OR=coef(fit_B), confint(fit_B)))

# ══ 4. INTERNAL VALIDATION (bootstrap) ═════
mod_B <- lrm(CPSP ~ catast + pain_hx + prior_surg +
   anxiety + RA + ketamine + ketoprofen + sev_pain +
   dn4_h48 + hyperalg + nefopam, data=dB, x=TRUE, y=TRUE)
val_B <- validate(mod_B, method='boot', B=1000)
brier <- mean((fitted(fit_B)-dB$CPSP)^2)

# ══ 5. MEDIATION ANALYSIS ═══════════════════
d1 <- complete(imp, 1)
m_mod <- glm(sev_pain ~ RA + age + bmi + Stade +
   chirurgie + catast + HADS_D + ICBN, family=binomial, data=d1)
y_mod <- glm(CPSP ~ RA + sev_pain + RA:sev_pain +
   age + bmi + Stade + chirurgie + catast + HADS_D + ICBN,
   family=binomial, data=d1)
med_RA <- mediate(m_mod, y_mod, treat='RA',
   mediator='sev_pain', sims=1000, boot=TRUE,
   boot.ci.type='perc')

# ══ 6. E-VALUES ═══════════════════════════
evalueOR(est=0.82, lo=0.53, hi=1.27, rare=FALSE, true=1)    # RA NDE
evalueOR(est=0.80, lo=0.55, hi=1.17, rare=FALSE, true=1)    # Ketamine NDE
```

R version 4.3.x (R Core Team, Vienna, Austria). Full code available from the corresponding author on reasonable request.
